# Effects of COVID‐19 lockdown on eating disorders and obesity: A systematic review and meta‐analysis

**DOI:** 10.1002/erv.2861

**Published:** 2021-08-30

**Authors:** Lucia Sideli, Gianluca Lo Coco, Rubinia Celeste Bonfanti, Bianca Borsarini, Lucia Fortunato, Cristina Sechi, Nadia Micali

**Affiliations:** ^1^ Department of Human Science LUMSA University Rome Italy; ^2^ Department of Psychology, Educational Science and Human Movement University of Palermo Palermo Italy; ^3^ Department of Psychiatry Faculty of Medicine University of Geneva Geneva Switzerland; ^4^ Department of Pedagogy, Psychology, Philosophy University of Cagliari Cagliari Italy

**Keywords:** COVID‐19, distress, eating disorders, meta‐analysis, obesity, systematic review

## Abstract

**Objective:**

This systematic review and meta‐analysis aimed to examine: the pooled prevalence of symptomatic behaviours and mental health deterioration amongst individuals with eating disorders (EDs) and obesity during the COVID‐19 confinement. Moreover, we examined changes in EDs and distress before and during the confinement, and the association between psychosocial factors and EDs symptoms.

**Method:**

A systematic search was carried out in biomedical databases from January 2020 to January 2021. Both cross‐sectional and longitudinal studies that used quantitative measures of ED symptoms and psychological distress during and after the COVID‐19 confinement were included.

**Results:**

A total of 26 studies met inclusion criteria (*n* = 3399, 85.7% female). The pooled prevalence of symptomatic deterioration in EDs was 65% (95% CI[48,81], *k* = 10). The pooled prevalence of increased weight in obesity was 52% (95% CI[25,78], *k* = 4). More than half of the participants experienced depression and anxiety. Moreover, at least 75% of the individuals with EDs reported shape and eating concerns, and increased thinking about exercising. However, the pooled analyses of longitudinal studies showed no significant differences from pre‐pandemic levels to the first lockdown phase in Body Mass Index and ED symptoms, whereas only few studies suggested increased distress, particularly among individuals with anorexia nervosa.

**Conclusions:**

The majority of individuals with EDs and obesity reported symptomatic worsening during the lockdown. However, further longitudinal studies are needed to identify vulnerable groups, as well as the long‐term consequences of COVID‐19.

## Key points

- Sixty‐five percent of the individuals with Eating Disorders experienced symptom deterioration during the COVID‐19 confinement
- Fifty‐two percent of the individuals with obesity reported weight increase
- More than half of the participants experienced depression and anxiety
- However, the few studies that examined changes in symptoms before and during the confinement showed inconsistent findings
- High‐quality longitudinal studies are needed to identify vulnerable groups, as well as the long‐term impact of COVID‐19

**Abbreviations**
AN: anorexia nervosa
BED: Binge Eating Disorder
BMI: Body Mass Index
BN: Bulimia Nervosa
DASS: Depression Anxiety Stress Scales
DERS‐SF: Difficulties in Emotion Regulation Scale‐Short Form
EDE‐Q: Eating Disorder Examination Questionnaire
EDI‐2: Eating Disorder Inventory‐2
EDs: Eating Disorders
GAD: Generalized Anxiety Disorder Scale
OSFED: other specified feeding and eating disorder
PHQ: Patient Health Questionnaire
UFED: unspecified feeding and eating disorder

## 1 INTRODUCTION

Coronavirus infection (COVID‐19) has become a complex public health problem since the beginning of 2020. Quarantine and social distancing were considered the most helpful measures in containing the infection (WHO, 2020) and most countries issued varying degrees of ‘shelter‐in‐place’ orders to slow down the spread of COVID‐19 during the first waves of the pandemic. However, there is evidence that undergoing lockdown can have detrimental effects on people's psychological health (Brooks et al., 2020). Consequently, during the first wave of the COVID‐19 pandemic and the first phase of lockdowns, it was recognised that the research on the COVID‐19 pandemic effects on health would need to be addressed as a priority (Holmes et al., 2020).

There is evidence of the negative consequences of the pandemic on individuals' psychological distress both in general population (Xiong et al., 2020; Wu et al., 2021) and in COVID‐19 patients (Taquet et al., 2021) during the lockdown phase in the spring/summer 2020. More specifically, longitudinal evidence suggests that in the first semester of the pandemic, mental health (i.e., anxiety, depression, and general distress) had deteriorated compared with the time before the pandemic (i.e., pre‐COVID 19) (Pierce et al., 2020; Prati & Mancini, 2021). Short‐term mental health consequences of COVID‐19 were equally high across affected countries, particularly among women, young adults (Cènat et al., 2021; Niedzwiedz et al., 2021), individuals belonging to lower socio‐economic status and healthcare workers (Krishnamoorthy et al., 2020; Luo et al., 2020). However, subsequently to the ease of social restrictions after lockdown, mental health problems decreased slightly but failed to reach the same level of pre‐COVID‐19 pandemic (Richter et al., 2021). To date limited research efforts have investigated a broad range of effects of the confinement on pre‐existing clinical groups (Rodgers et al., 2020; Shah et al., 2020).

It was argued that the COVID‐19 lockdown can affect eating disorders (EDs), by triggering EDs behaviours and exacerbate existing symptoms. Potential risk pathways include: restriction to daily activities and movements, excessive exposure to harmful eating patterns on social media, emotional distress, fear of contagion, and low access to treatment and care (Rodgers et al., 2020). The confinement might also increase psychological suffering among individuals with obesity (Almandoz et al., 2020; Athanasiadis et al., 2020, Marchitelli et al., 2020; Sisto et al., 2020). Considering the multiple disruptive consequences of the COVID‐19 lockdown on individuals with EDs (Rodgers et al., 2020), especially during the first waves of the pandemic, home confinement might analogously have a negative impact on weight management and weight gain, both important issues for individuals with obesity (Bhutani & Cooper, 2020). Indeed, social restrictions, changes in lifestyle and reduction in exercise practice can lead to a worsening in the nutritional and metabolic status of individuals with obesity (Kwok et al., 2020). On the basis of the repercussions of the COVID‐19 confinement on weight status, eating behaviour, physical activity, body shape and perception, we decided to consider both EDs and obesity together in our study.

Only few studies have examined the effects of the COVID‐19 confinement on individuals with EDs, showing mixed findings (Baenas et al., 2020; Branley‐Bell & Talbot, 2020; Fernández‐Aranda, Munguía et al., 2020; Giel et al., 2021; Graell et al., 2020; Monteleone, Cascino, et al., 2021; Schlegl, Maier, et al., 2020; Termorshuizen et al., 2020). Although most of these previous studies reported heightened levels of ED symptoms and psychological distress in individuals with EDs, positive and negative impacts of restrictive measures, especially during the initial lockdown phase, might depend on the ED subtype. For example, some studies showed a higher risk of deterioration in the individuals with anorexia nervosa (AN) (Baenas et al., 2020; Schlegl, Maier, et al., 2020; Termorshuizen et al., 2020), whereas other studies reported limited impact of lockdown in both individuals with AN and obesity (Fernández‐Aranda, Munguía et al., 2020). Moreover, the persistence of a deterioration in clinical symptoms after lockdown, as well as the long‐term effect of the confinement on mental health, is largely unknown in individuals with EDs and obesity (Clemmensen et al., 2020; Monteleone, Marciello, et al., 2021).

The aim of the present systematic review and meta‐analysis is to analyse the impact of the COVID‐19 confinement on mental health of the individuals with EDs and obesity. Specifically, the current study aims to: analyse the pooled prevalence of both ED symptoms and mental health deterioration; examine differences in the prevalence of symptoms among controls compared to individuals with EDs or obesity; examine changes in EDs and mental health symptoms before and during the confinement. Finally, we examined the association between psychosocial factors and ED symptoms during the confinement, to identify potential predictors of distress. Summarising the evidence on psychopathological symptoms among both the participants with EDs or obesity cannot only shed light on how this specific clinical group coped with the global confinement, but it can also impact on the therapeutic strategies and health care service provision for EDs.

## 2 METHODS

### 2.1 Search strategy and eligibility criteria

The review and meta‐analysis were conducted according to the preferred reporting items for systematic reviews and meta‐analyses (PRISMA) statement (Moher et al., 2015). All empirical studies that investigated individuals with eating disorders or obesity as a primary diagnosis during the COVID‐19 confinement were eligible, with the following inclusion criteria: (1) original articles, (2) written in English, (3) having a cross‐sectional, case‐control, cohort, or mixed‐method study design and (4) assessing eating symptoms and psychopathology of EDs and obesity during the COVID‐19 confinement. The primary outcome was eating disorder psychopathology, and secondary outcomes were mental health symptoms such as depression, anxiety, general distress, post‐traumatic stress disorder, sleep disturbances, and negative affect. Publications were excluded if (1) they were not original articles (e.g., conference paper, proceeding, review, opinion paper, dissertation, case series or case report), and (2) the population studied did not include EDs or obesity.

A systematic and comprehensive search was performed using the following databases: PubMed/Medline, ISI Web of Science, PsychInfo, EMBASE, SCOPUS and Google Scholar. Moreover, Internet searches on pre‐print servers for unpublished papers were adopted. Searches were limited to studies published from January 2020 to January 2021. Search terms employed were (Obesity OR overweight OR weight gain OR anorexia* OR bulimia* OR binge eating disorder* OR feeding OR malnutrition OR avoidant restrictive food intake disorder (ARFID) OR pica OR rumination OR other specified feeding and eating disorder (OSFED) OR unspecified feeding and eating disorder (UFED) AND (COVID OR COVID 19 OR pandemic* OR SARSCoV2 OR coronavirus disease OR coronavirus OR HCoV).

### 2.2 Data extraction and analyses

Lucia Sideli, Bianca Borsarini, Lucia Fortunato, Cristina Sechi, and Rubinia Celeste Bonfanti extracted data from the eligible studies into a customised Excel spreadsheet. The following information from each study was extracted: authors, year of publication, country, and sample characteristics (sample size, diagnosis, mean age, percentage female, type of outcome measure, follow‐up period, and study results). At this stage, quality checks were iterative consultation of data and, careful cross‐checking of the extracted data, and consensus decisions about methodology.

A meta‐analysis was conducted to assess the overall prevalence of deterioration of relevant outcomes among the EDs and the obesity groups, change in ED symptoms and mental distress from pre‐pandemic baseline levels over the course of the confinement. Only outcomes with data available from at least *k* = 3 studies or samples were included. When available, study findings related to specific EDs were included. Odds ratios (*ORs*) with 95% confidence interval (95% CI) were calculated to estimate the pooled prevalence using random‐effects model with the metaprop Stata routine. Cohen's *d* with 95% CI for comparisons between groups with equal size was calculated to estimate the average change between pre‐ and during the confinement using the meta Stata routine. Heterogeneity was assessed using I‐squared (*I* ^2^) statistics, assuming 0%–25%, 25%–50%, and 50%–75% *I* ^2^ values corresponding to low, moderate, and high heterogeneity, respectively. For *k* ≥ 5, formal assessment of publication bias was carried out. Doi plot and LFK index were used to explore publication bias in prevalence studies, with LFK index > |1| suggesting minor asymmetry and >|2| suggesting major asymmetry (Furuya‐Kanamori et al., 2018). Funnel plot and Egger's regression test were used to assess publication bias in studies concerning changes before and during the confinement. Statistical significance for Egger's test was set at *p* < .05. Meta‐analysis was carried out using Stata 16 (Press, 2019).

### 2.3 Quality assessment

A quality appraisal was carried out using a modified version of the Newcastle‐Ottawa Scale (Wells et al., online) for observational studies (see Tables S1 and S2). A maximum of nine points were attributed. Studies were evaluated to be at low risk of bias if scored seven to nine, at moderate risk of bias if scored five or six, and at high risk of bias if scored equal or lower than four. Quality assessment was conducted by Bianca Borsarini, Cristina Sechi, Lucia Fortunato, Lucia Sideli, and Rubinia Celeste Bonfanti. Any discrepancies between reviewers were discussed until an agreement was reached, if needed on the senior authors were consulted (Gianluca Lo Coco and Nadia Micali).

## 3 RESULTS

**FIGURE 1:**
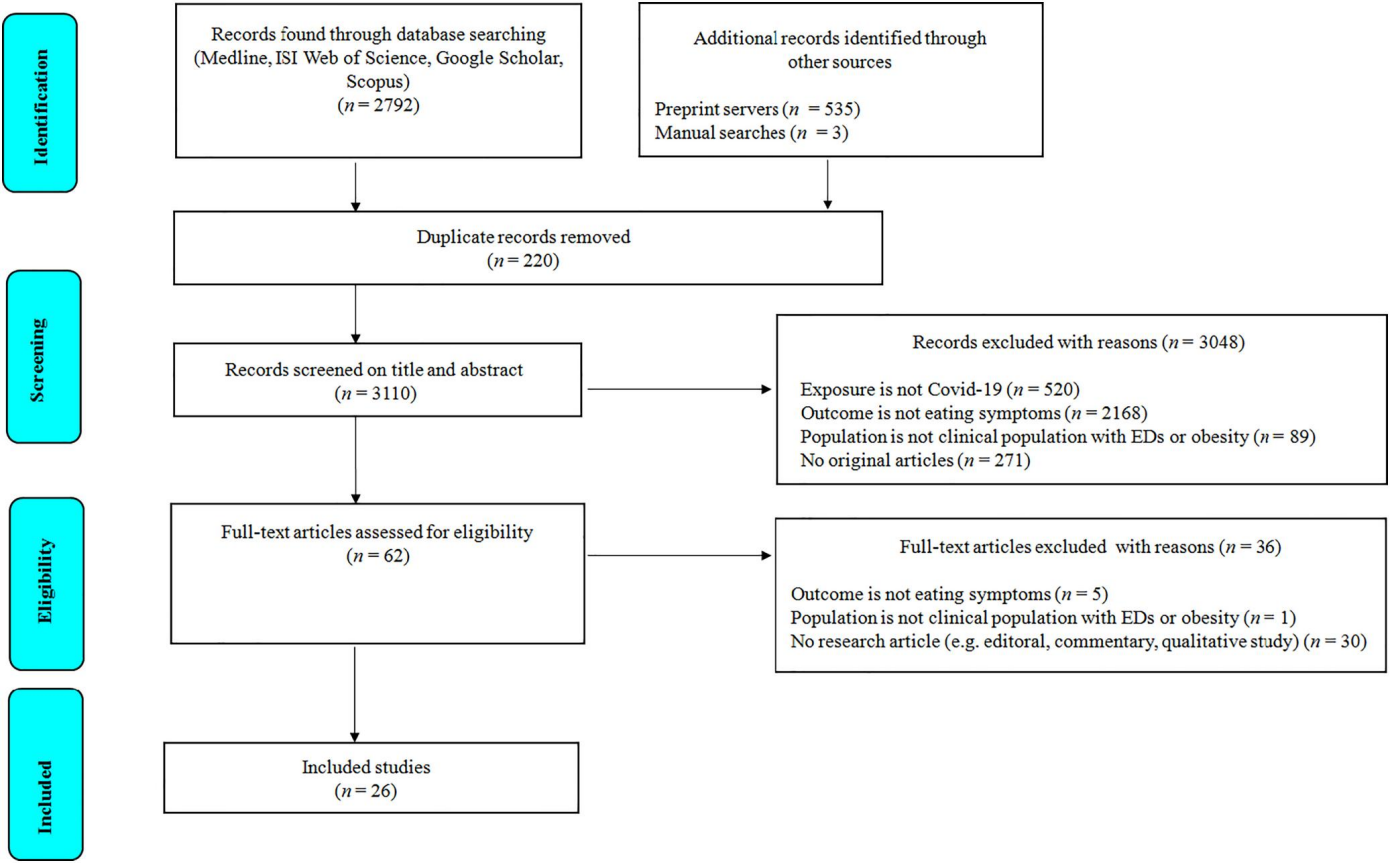
Preferred reporting items for systematic reviews and meta‐analyses (PRISMA) flowchart of study selection

Figure 1 provides the flow diagram of all articles retrieved and included in the systematic review. A total of 3327 hits from databases, pre‐print servers, and manual searches of references were returned. Following removal of 220 duplicates, 3110 remaining titles and abstracts were screened, and 62 full‐text articles were assessed for eligibility. The 26 articles that met inclusion criteria for the systematic review comprised 16 studies focused on EDs, 9 on clinical obesity, and 1 on both EDs and obesity (see Figure 1).

### 3.1 Quality appraisal

Table S1 presents the quality ratings of included studies. Overall, 23% of the studies (18% EDs and 33% obesity studies) fully satisfied the criteria for robustness. Thirty‐five percent (29% EDs and 55% obesity studies) were evaluated at medium risk of bias, and a further 42% (53% EDs and 22% obesity studies) at high risk of bias. Nineteen percent of the studies adopted a mixed‐method design by including also qualitative data. Only 15% of studies provided longitudinal data; and 19% of studies (4 EDs and 1 obesity studies) compared individuals with EDs with healthy controls. Most studies reported a high selection bias, with 35% of the studies using inadequate recruitment strategies (e.g., snowballing recruitment of individuals with self‐reported EDs), and 88% reporting inadequate or unspecified participation rate.

The majority of the studies (62%) used a validated measure of EDs and/or mental health symptoms, such as the Eating Disorder Examination Questionnaire (EDE‐Q; Fairburn & Beglin, 1994), the Eating Disorder Inventory‐2 (EDI‐2; Garner, 1991), the Generalized Anxiety Disorder Scale (GAD; Spitzer et al., 2006), the Depression Anxiety Stress Scales (DASS; Lovibond & Lovibond, 1995), the Difficulties in Emotion Regulation Scale‐SF (DERS‐SF; Kaufman et al., 2016), and the Patient Health Questionnaire (PHQ; Kroenke et al., 2001). The majority of the studies (76%) provided complete information on the statistical analyses used (e.g., statistical tests and 95% CI or *p* value). Twenty‐seven percent controlled the analysis for basic socio‐demographic variables and another 31% for additional potential confounders (e.g., ED symptoms or diagnosis, or Body Max Index (BMI), or mood symptoms).

### 3.2 Eating disorders

#### 3.2.1 Characteristics of the studies

The characteristics of included studies are presented in Table S3. Results are divided into four sections, according to type of outcome (i.e., (a) studies examining the prevalence of symptom deterioration during the confinement, followed by (b) studies examining symptom's change from pre‐pandemic to confinement time, (c) the comparison between cases and controls during the confinement, and (d) studies examining the association between psychosocial factors and ED symptoms). Seventeen studies on EDs reported symptoms which were assessed between April and June 2020 from both European (Germany, Ireland, Italy, Netherlands, Spain, Portugal, and UK) and non‐European countries (Australia, Canada, and US). Most of the studies involved adult participants, three studies (Schlegl, Maier, et al., 2020, Schlegl, Meule, et al., 2020; Termorshuizen et al., 2020) included both adult and adolescent participants, and only one study (Graell et al., 2020) included children and adolescents. The total number of participants was 3399, with 85.7% of females (*n* = 2912) and a mean age of 26.7. Limiting the studies to those with adolescents and adults, the total participants were 3,034 (*n* = 2,661, 87.7% of females (range 63%–100%), mean age 29.1 (range 22.4–36.8), mean BMI 22.6 (range 17.3–33.5), comprising 49.4% (*n* = 1499) of individuals with AN, 20.6% (*n* = 624) with bulimia nervosa (BN), 12.4% (*n* = 375) with binge eating disorder (BED), and 33.4% (*n* = 1,013) with other specified feeding or eating disorders (OSFED) or unspecified EDs, with several studies including participants with multiple EDs diagnoses. Nine study samples were selected among former or current service users of EDs inpatient or outpatient services (Baenas et al., 2020; Castellini et al., 2020; Fernández‐Aranda, Munguía et al., 2020; Graell et al., 2020; Machado et al., 2020; Monteleone, Cascino, et al., 2021, Monteleone, Marciello, et al., 2021; Richardson et al., 2020; Schlegl, Maier, et al., 2020, Schlegl, Meule, et al., 2020), and only a few provided information about disease or treatment status at the time of the study, suggesting that the majority of the participants had a current symptomatic EDs and/or were in psychological or combined treatment (Baenas et al., 2020; Castellini et al., 2020; Fernández‐Aranda, Munguía et al., 2020; Graell et al., 2020; Machado et al., 2020; Monteleone, Cascino, et al., 2021, Monteleone, Marciello, et al., 2021). Among the studies involving individuals with self‐reported EDs (Branley‐Bell & Talbot, 2020; McNamara et al., 2020; Phillipou et al., 2020; Quittkat et al., 2019; Robertson et al., 2021; Termorshuizen et al., 2020; Vuillier et al., 2021), four studies suggested that the proportion with symptomatic ED or under specialised treatment ranged from 20% to 93% (Branley‐Bell & Talbot, 2020; McNamara et al., 2020; Termorshuizen et al., 2020; Vuillier et al., 2021).

### 3.3 Prevalence of ED deterioration and mental health symptoms during the confinement

**FIGURE 2:**
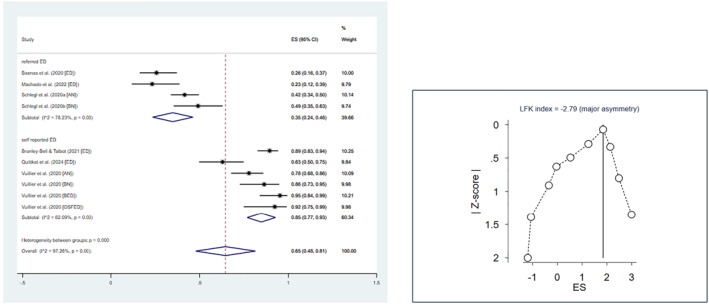
Forest and Doi plot of symptom deterioration in eating disorders. 95% CI = 95% confidence interval; EDs = eating disorders; ES = effect size

**FIGURE 3:**
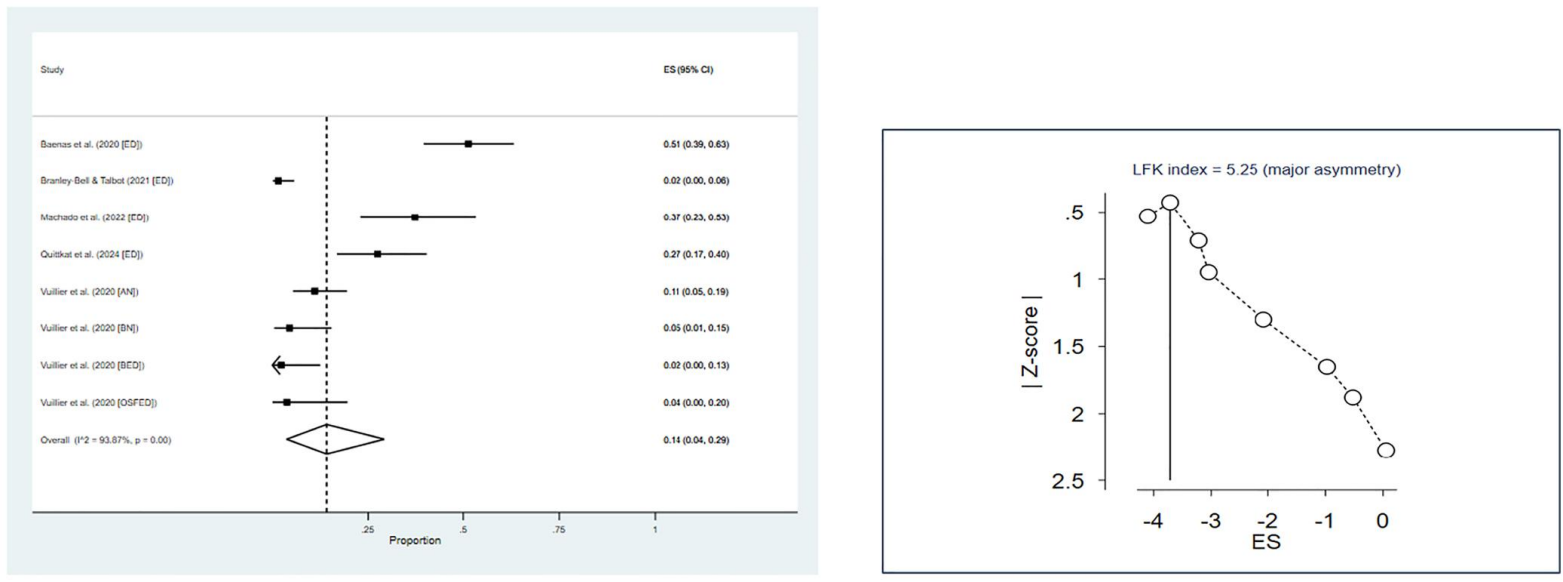
Forest and Doi plot of symptom improvement in eating disorders. 95% CI = 95% confidence interval; EDs = eating disorders; ES = effect size

Among adolescent and adult samples, the prevalence of symptom deterioration during the confinement was 65% (Table 1). Heterogeneity analyses revealed greater prevalence in studies of individuals with self‐reported ED compared to patients with ED (Figure 2) and no difference between medium‐ and high‐quality studies (heterogeneity between sub‐groups (1) = 0.11, *p* = 0.74). Only 16% of the pooled sample reported ED symptoms being less present or improving during the confinement (Table 1; Figure 3). Doi plot and LFK index suggested potential publication bias for both worsening (LFK = −2.79) and improvement (LFK = 5.25), in opposite directions. During the confinement, at least 75% of individuals with EDs reported shape concerns, eating concerns, and increased thinking about exercising. Restricting behaviours and increased exercising were reported by 60% and by 44% of the pooled sample, binge eating by 32%, and purging by 12% (Table 1). Negative publication bias was suggested for restricting behaviours (LFK = −2.80). As far as mental health symptoms were concerned, more than a half of the participants experienced depression and anxiety, and about one third of participants complained about the reduced quality of therapy. A single study on children and adolescents found that 41.9% experienced symptom worsening during lockdown, and 37% general psychopathology symptoms (Graell et al., 2020).

**TABLE 1:** Prevalence of eating‐related and mental health symptoms among individuals with eating disorders

	Eating‐related symptoms				*p*	*I* ^2^ (%)
Domain	*k*	Random pooled ES	95% CI	Heterogeneity *χ* ^2^		
Symptom worsening	10	0.65	0.48; 0.81	329.03	<0.001	97.26
Symptom improvement	8	0.16	0.07; 0.24	111.90	<0.001	93.74
Shape concerns	3	0.81	0.62; 1.00	36.35	<0.001	94.50
Eating concerns	3	0.79	0.73; 0.86	3.0	0.22	33.81
Increased thinking about exercising	4	0.76	0.68; 0.84	11.91	0.01	74.81
Restricting behaviour	5	0.60	0.49; 0.71	68.28	<0.001	94.14
Increased exercising	5	0.44	0.36; 0.52	12.86	0.01	68.90
Binging	5	0.32	0.19; 0.44	100.04	<0.001	96.00
Purging	3	0.12	0.04; 0.19	11.57	<0.001	82.71

Mental health symptoms						
Domain	*k*	Random pooled ES	95% CI	Heterogeneity *χ* ^2^	*p*	*I* ^2^ (%)
Depression	4	0.55	0.39; 0.70	35.71	<0.001	91.60
Anxiety	4	0.50	0.42; 0.57	8.02	0.05	62.58
Reduced quality of therapy	4	0.49	0.27; 0.70	109.64	0.001	97.26

*Note*: CI, confidence interval; EDs, eating disorders; ES, effect size.

#### 3.3.1 Change in symptoms before and during the confinement

No significant changes in BMI, ED symptom severity, impact of ED symptoms, and binge eating from pre‐pandemic baseline levels over the course of the confinement were found (Figures S1–S5). Furthermore, no change in mental health symptoms was found (Figures S6 and S7). No evidence of publication bias was found, neither for BMI (Egger's test *z* = −0.33, *p = *.378) nor for mental health symptoms (*z* = 0.89, *p* = .371) (Figures S1, S2, S6 and S7).

Concerning other ED‐related and psychopathological outcomes with less than three studies, no increase in physical exercise was found in two clinical samples of patients with AN and BN (Castellini et al., 2020) and a sample with mixed EDs (Monteleone, Cascino, et al., 2021). One study reported increased levels of anxiety, stress, insomnia, suicide, and isolation during lockdown compared to before the pandemic (Monteleone, Cascino, et al., 2021, Monteleone, Marciello, et al., 2021). Individuals with AN showed greater level of anxiety and suicide compared to those with other EDs (Monteleone, Marciello, et al., 2021). Another study reported higher psychosocial stress during lockdown than before the pandemic among individuals with self‐reported EDs (Quittkat et al., 2019). Moreover, results on emotion dysregulation were inconsistent (Fernández‐Aranda, Munguía et al., 2020; Machado et al., 2020), with evidence of any change reported only among individuals with AN (Fernández‐Aranda, Munguía et al., 2020). Finally, a single study compared the levels of EDs and psychopathological symptoms between two samples of individuals contacting a national ED helpline in March–April 2019 and in March–April 2020. An increased frequency of restricting behaviours, increased physical exercise, purging, anxiety, and depression was identified (Richardson et al., 2020).

### 3.4 Comparison between cases and controls during the confinement

Three studies compared ED cases with healthy controls during the confinement. Compared to healthy controls, a mixed clinical sample of individuals with EDs reported increased binge eating, compensatory physical exercise, and stress (Castellini et al., 2020). Quittkat et al. (2019) found that individuals with self‐reported EDs experienced greater levels of stress compared to healthy controls. In the study by Robertson et al. (2021), individuals with self‐reported ED presented a greater difficulty to control eating behaviours, greater concern about body shape, increased thinking, and practice of physical activity. Furthermore, they experienced more preoccupation about food compared to both healthy controls and individuals with self‐reported other mental disorders (Robertson et al., 2021).

### 3.5 Predictors of ED symptoms

Seven studies examined the relationship between EDs and psychosocial factors. Isolation positively correlated with ED symptoms among both patients and individuals with self‐reported EDs (McNamara et al., 2020; Monteleone, Marciello, et al., 2021) but not with changes in physical activity (Vuillier et al., 2021). Perceived social support was higher among recovered patients, compared to those with a current disorder (Branley‐Bell & Talbot, 2020). COVID‐19‐related stress and disruption to routine predicted symptom worsening (Baenas et al., 2020), overall symptom severity (Machado et al., 2020; McNamara et al., 2020; Monteleone, Marciello, et al., 2021), specific ED symptoms, such as binge eating (Castellini et al., 2020), eating and body concerns (Robertson et al., 2021), and, inconsistently, exercising (Robertson et al., 2021; Vuillier et al., 2021). Furthermore, COVID‐19‐related stress predicted ED‐related impairment, also via emotion dysregulation (Machado et al., 2020). Although no effect of general psychopathology on ED worsening was found in one study (Baenas et al., 2020), other studies suggested a relationship between anxiety and ED symptoms (McNamara et al., 2020), between negative emotions and change in physical exercising in AN (Vuillier et al., 2021), and between emotional dysregulation and ED symptoms in BN (Vuillier et al., 2021).

One study identified self‐directedness as a protective factor for worsening of ED (Baenas et al., 2020), another found that perceived control was higher among patients recovered from EDs compared to symptomatic patients (Branley‐Bell & Talbot, 2020), and a third that difficulty in maintaining goals when upset predicted ED symptom severity across different diagnoses (Vuillier et al., 2021). Exposure to triggering messages and change in food availability positively correlated with change in physical activity among patients with ED, and especially among those with AN and OSFED (Vuillier et al., 2021). Finally, quality of family relationships was found to be associated with severity of overall (McNamara et al., 2020) or specific ED symptoms (Castellini et al., 2020).

### 3.6 Obesity

#### 3.6.1 Characteristics of the studies

The 10 studies on obesity were based on adult (*n* = 9) and children (*n* = 1) samples (Abawi et al., 2020) from Italy, Iraq, Netherlands, Poland, Spain, UK, and US, assessed between April and July 2020 (Table S4). The 2718 adult participants were mostly females (*n* = 1,973, 72.6%, range 39.5%–87%), with a mean age of 46.2 (range 33.5–51.2 years) and a mean BMI of 39.2 (range 34.4–41.2). Obesity severity was unspecified for all but one of the studies (Marchitelli et al., 2020). All participants were service users of bariatric or obesity clinic.

### 3.7 Prevalence of eating and mental health symptoms

More than 50% of the individuals with obesity reported increased weight during lockdown, reduced levels of physical activity, and increased snacking (Table 2). Only two studies reported increased difficulties to follow a diet (range 49%–70%; Almandoz et al., 2020; Sisto et al., 2020) and more frequent binge eating (range 19%–45%; Athanasiadis et al., 2020; Marchitelli et al., 2020).

**TABLE 2:** Prevalence of eating disorders and mental health symptoms among individuals with obesity

	Eating‐related symptoms				*p*	*I* ^2^ (%)
Domain	*k*	Random pooled ES	95% CI	Heterogeneity *χ* ^2^		
Increased weight	4	0.52	0.25; 0.78	355.16	<0.001	99.16
Reduced physical activity	4	0.61	0.50; 0.72	38.67	<0.001	92.24
Increased snacking	4	0.56	0.49; 0.63	10.83	0.01	72.31

Mental health symptoms						
Domain	*k*	Random pooled ES	95% CI	Heterogeneity *χ* ^2^	*p*	*I* ^2^ (%)
Depression	3	0.51	0.18; 0.85	193.25	<0.001	98.97
Anxiety	4	0.51	0.21; 0.81	451.31	<0.001	99.34
Loneliness	3	0.31	0.15; 0.46	72.94	<0.001	97.26

*Note*: CI, confidence interval; EDs, eating disorders; ES, effect size.

A single study on children with obesity found that about one‐third of participants (32%) reported anxiety (Abawi et al., 2020). Across adolescent and adult samples, the prevalence of psychopathology was about 50% for both anxiety and depression, whereas only 31% reported loneliness (Table 2).

### 3.8 Change in symptoms before and during the confinement

Three studies assessed obesity‐related and psychological outcomes either using independently collected or retrospectively assessed measures. One study found a mild increase in weight and BMI (Pellegrini et al., 2020), while an other study reported a mild decrease (Fernández‐Aranda, Munguía et al., 2020). No significant changes were observed in anxiety/depression, emotion regulation, and COVID‐19 impact on ED symptoms (Fernández‐Aranda, Munguía et al., 2020). A single study on children and adolescents found no difference in quality of life and emotional functioning between pre‐pandemic and the confinement (Abawi et al., 2020).

### 3.9 Comparison between cases and controls during the confinement

A single case‐control study found that individuals with self‐reported obesity experienced greater weight gain compared to those at normal weight, as well as increased eating and snacking (Sidor & Rzymski, 2020).

### 3.10 Predictors of symptoms

Anxiety and depression were associated with increased weight and BMI in one study (Pellegrini et al., 2020). However, no effect of feelings or boredom/solitude was found on weight gain or BMI (Pellegrini et al., 2020) during the confinement. Another study showed that anxiety contributed to weight gain in individuals with obesity without other mental disorders, while depression exerted the opposite effect (Marchitelli et al., 2020). In the same study, anxiety and depression did not predict weight gain among individuals with obesity with comorbid psychiatric diagnoses (Marchitelli et al., 2020). In a sample of post‐bariatric individuals with obesity, depression, anxiety, and stress were associated with increased hunger, impulsivity in eating, and snacking, whereas fear of COVID‐19 was not (Sisto et al., 2020).

## 4 DISCUSSION

### 4.1 Effect of the pandemic on eating disorders and obesity

The present meta‐analysis of 26 studies (16 with 3399 participants suffering from EDs and 10 with 2718 individuals with obesity) analysed the prevalence of EDs and mental health symptom deterioration during the COVID‐19 confinement, changes in EDs and mental health symptoms before and during lockdown, and the association between psychosocial factors and ED symptoms. According to available findings, 65% of individuals with EDs experienced worsening of symptoms during the confinement, whereas only 16% reported mild or moderate improvement. Overall, more than 75% of individuals with EDs experienced shape concerns, eating concerns, and increased thinking about exercising. Furthermore, more than 50% of individuals with obesity reported increased weight, snacking, and reduced physical activity. Anxiety and depression were common in the two populations and affected at least 50% of individuals.

The few case‐control studies included suggested that patients with EDs and individuals with self‐reported EDs or obesity experienced increased ED symptoms and higher stress compared to healthy controls. However, pooled analyses of studies that examined changes in symptoms before and during the confinement showed mixed findings. We did not find significant differences in BMI, ED symptom severity, impact of ED symptoms, and binge eating, whereas only sporadic studies suggested increased mental health symptoms, particularly among individuals with AN. It is worth noting that this analysis was run on a very limited subset of studies (15% of the eligible papers), making it difficult to assess changes in symptoms and distress from pre‐pandemic baseline levels over the course of the lockdown.

### 4.2 Comparison with previous reviews

Earlier narrative reviews published in the late 2020 (Moreno et al., 2020; Pedrosa et al., 2020) pointed out that individuals with pre‐existing mental disorders, including EDs, were at increased risk for symptom re‐activation as well as for depression, anxiety, insomnia, and post‐traumatic stress. The findings of this review confirm these preliminary data and provide estimates of the burden of the first lockdown phase (between April and June 2020) on individuals with EDs and clinical obesity, which may contribute to target future interventions and to inform healthcare policies. However, the low but significant proportion of individuals reporting mild to moderate improvement as well as the inconsistent findings on changes in symptoms before and during the confinement may suggest that the negative effect of the COVID‐19 pandemic was highly heterogeneous. This is consistent with a meta‐analysis of longitudinal general population studies, which found that COVID‐19 lockdown significantly increased the risk of depression and anxiety, but not those of general distress, negative affect, and suicide (Prati & Mancini, 2021). In that meta‐analysis, heterogeneity among studies was not explained either by the main demographic or COVID‐19‐related variables (i.e., COVID‐19 death rate and days of lockdown). Moreover, large longitudinal studies suggested that distress levels declined quickly after an initial rise at the start of the pandemic (Daly & Robinson, 2021). Thus, more research is needed to examine temporal changes in mental health symptoms of both individuals with EDs and individuals with obesity.

### 4.3 Vulnerable populations

There is evidence that increases in mental distress did not affect all groups equally (Pierce et al., 2020). History of mental health problems, loneliness, external locus of control, and higher levels of intolerance to uncertainty and death anxiety have been also associated with increased psychological distress (Shevlin et al., 2021). In our review, the few studies (19% of the eligible papers) comparing EDs or obesity cases with healthy controls during the confinement showed higher symptomatic behaviours and mental distress, suggesting that individuals with eating or weight disorders can be more at risk. However, none of these studies controlled for pre‐pandemic symptoms and distress, thus providing only a preliminary level of evidence.

In the present review, subgroup analyses indicated that symptom worsening was more prevalent among individuals with self‐reported EDs than patients attending ED clinical services. This finding might suggest that individuals with self‐reported EDs were more likely to overestimate their ED symptoms (i.e., recall bias). However, it is also possible that patient contact with clinical services contributed to a reduction of the impact of the pandemic on the course of EDs, through the implementation of online consultations and support for both previous and recently referred patients (Fernández‐Aranda, Casas, et al., 2020). Indeed, Monteleone, Cascino, et al., 2021 found that the quality of perceived therapeutic relationship was one of the most important protective factors towards symptom deterioration. Furthermore, it is likely that individuals who had any treatment prior to the pandemic compared to individuals who never received therapy were able to cope with the distressing impact of the COVID‐19 lockdown, both on EDs and general psychopathology, by using the skills learnt during their therapy (e.g., emotion regulation). However, given the few number of studies, further investigations in this area are needed.

### 4.4 Risk and protective factors

Regarding the correlates of ED symptoms, our results suggest that social isolation and COVID‐19‐related stress were frequently associated with ED worsening and weight gain. On the contrary, family and social support seem to exert a protective role. Although most studies suggested that anxiety, depression, and negative emotions were related to EDs symptoms, findings were not consistent amongst individuals with obesity. However, only three studies examined the association between weight gain and psychosocial distress among the population with obesity preventing firm conclusions. Moreover, given that the reviewed studies were carried out during the first lockdown phase, it is possible that these factors show a detrimental role in the long term.

Furthermore, three studies observed that self‐directedness, perceived control, and capacity to maintain goals were associated with recovery or lack of deterioration of ED. It is likely that specific factors may play a more relevant role among specific clinical populations, for instance that unpredictability of the situation and lack of control greatly affected individuals with AN, whereas difficulties in emotion regulation affected binge eating episodes more strongly (Todisco & Donini, 2021). In line with these findings, Giel et al. (2021) showed that the use of cognitive reappraisal an emotion regulation strategy and sense of coherence were associated with reduced eating symptoms among patients with BED during the first COVID‐19 lockdown.

The results of this review suggests that the role of broader social risk factors, such as exposure to triggering and potentially stigmatising messages (e.g., mass media communication about weight gain during quarantine, the so called ‘quarantine‐15’) have been poorly investigated. Along the same lines, future research should examine the positive role of social media in allowing individuals with EDs to stay in touch with their friends and getting support from ED online communities, thus buffering the negative consequences of home‐confinement (Branley‐Bell & Talbot, 2020; Rodgers et al., 2020). Overall, the current review highlights the need of further research efforts to identify specific vulnerability and protective factors among individuals with ED during the confinement.

### 4.5 Clinical implications

The findings of the current systematic review have some relevant clinical implications. One of the major challenges faced by individuals with EDs and obesity during confinement was the worsening of their clinical condition, associated with a reduction in healthcare service provision (Cooper et al., 2020). A significant number of vulnerable individuals belonging to these clinical populations were abruptly left with no access to healthcare services (Branley‐Bell & Talbot, 2020; Richardson et al., 2020) or had a difficult transition from face‐to‐face to online treatments (Lewis et al., 2021). Discontinued level of care was taxing (Richardson et al., 2020; Schlegl, Meule, et al., 2020) and our findings suggest that individuals with EDs who were not attending ED clinical services were at greater risk for symptom worsening. Thus, greater availability of treatment options, such as digital and e‐health ones may prove useful in this difficult time. Individuals with EDs who were not currently receiving psychotherapy are more likely to prefer e‐therapy (Linardon et al., 2020) and our findings further support the need of providing e‐health interventions to help individuals with EDs with limited access to care during the COVID‐19 confinement, or living in areas with limited ED and obesity service provision. Given the importance of facilitating access to care for new onset of symptoms, adapting standard care to the restrictions due to the pandemic has been recommended (Fernández‐Aranda, Casas et al., 2020; Weissman et al., 2020). Following 2 decades of research on digital assessment and treatment of EDs, several e‐health options for EDs range from online cognitive behavioural therapy (Murphy et al., 2020; Waller et al., 2020), to family therapy and online guided self‐help (Albano et al., 2021; Matheson et al., 2020). These digital treatments may provide support to patients during the pandemic, and this is especially important given that our findings suggested that social isolation and COVID‐19‐related stress may be associated with ED worsening and weight gain.

Furthermore, our findings suggest that self‐directedness, perceived control, and capacity to maintain goals were associated with lack of deterioration of EDs and there is evidence that these individual resources can be successfully addressed by online interventions. However, it is worth noting that although many patients appreciated the transition to telehealth during lockdown (Fernández‐Aranda, Munguía et al., 2020), several limitations of this treatment modality were raised, including inadequate Internet connectivity, lack of privacy, body image distress during tele‐therapy, and difficulties in managing weight self‐monitoring (Branley‐Bell & Talbot, 2020; Monteleone, Cascino, et al., 2021; Termorshuizen et al., 2020; Vuillier et al., 2021).

### 4.6 Limitations

There are several potential limitations to the current study. First, only a small number of longitudinal studies was available. Second, selected studies were carried out between February and June 2020, and we cannot examine the long‐term impact of the pandemic on symptoms and well‐being of individuals with EDs and obesity. Third, all the eligible studies mainly involved female participants. Although EDs are more prevalent in females, the generalisation of the findings to men may be limited. Fourth, the assessment of possible moderating factors was limited by the low number of studies, the heterogeneity of outcomes and study design, as well as by the lack of information about other potential moderating factors (e.g., ED diagnosis, obesity severity, BMI, illness duration, psychiatric comorbidities). Further studies should better explore these factors. Fifth, the assessment of publication bias was carried out only for a minority of ED‐related outcomes, because the others did not reach a minimum number of available studies. Preliminary evidence suggests a tendency to underreport some of the negative outcomes and over report improvement. This may suggest that studies detecting worsening of EDs might have been overlooked. However, no indication of publication bias was observed in studies comparing the condition of individuals with EDs before and during the confinement, as well as in those on individuals with obesity. A further limitation is the low quality of studies, with 77% of the included studies at moderate or high risk of bias. Future research should pay more attention to design longitudinal or case control studies, with lower selection bias and to control analyses for relevant socio‐demographic variables. Finally, no articles from Asia were included in the current meta‐analysis, and the generalisation of our findings may be limited.

## 5 CONCLUSIONS

This is the first systematic review and meta‐analysis reporting pooled prevalence estimates for ED and mental health symptoms among individuals with EDs and obesity during the COVID‐19 confinement. The findings show that two third of individuals with EDs experienced symptom deterioration during the confinement, and more than 50% of the individuals with obesity reported increased weight. However, the few studies that examined changes in symptoms before and during the confinement showed inconsistent findings. Overall, these results highlight the need for further high‐quality longitudinal studies that examine which clinical groups experienced higher distress than others, and whether the initial increase in symptoms after lockdown might represent a spike in distress response that stabilises or decreases as individuals adjust.

## CONFLICT OF INTEREST

The authors declare no conflict of interest.

## Supporting information

Supporting Information S1

## Data Availability

The data that support the findings of this study are available from the corresponding author upon reasonable request.

## References

[erv2861-bib-0001] Abawi, O. , Welling, M. S. , van den Eynde, E. , van Rossum, E. F. C. , Halberstadt, J. , van den Akker, E. L. T. , & van der Voorn, B. (2020). COVID‐19 related anxiety in children and adolescents with severe obesity: A mixed‐methods study. Clinical Obesity, 10(6), e12412. 10.1111/cob.12412 32920993 PMC7685119

[erv2861-bib-0003] Albano, G. , Cardi, V. , Kivlighan, D. M. , Ambwani, S. , TreasureLo Coco, J. G. , & Lo Coco, G. (2021). The relationship between working alliance with peer mentors and eating psychopathology in a digital 6‐week guided self‐help intervention for anorexia nervosa. International Journal of Eating Disorders, 54, 1–1526. 10.1002/eat.23559 PMC8453826 34042206

[erv2861-bib-0004] Almandoz, J. P. , Xie, L. Y. , Schellinger, J. N. , Mathew, M. S. , Gazda, C. , Ofori, A. , Kukreja, S. , & Messiah, S. E. (2020). Impact of COVID‐19 stay‐at‐home orders on weight‐related behaviours among patients with obesity. Clinical Obesity, 10(5), e12386. 10.1111/cob.12386 32515555 PMC7300461

[erv2861-bib-0005] Athanasiadis, D. I. , Hernandez, E. , Hilgendorf, W. , Roper, A. , Embry, M. , Don Selzer, M. D. , & Stefanidis, D. (2020). How are bariatric patients coping during the coronavirus disease 2019 (COVID‐19) pandemic? Analysis of factors known to cause weight regain among postoperative bariatric patients. Surgery for Obesity and Related Diseases, 17, 756–764. 10.1016/j.soard.2020.11.021 33390351 PMC7699156

[erv2861-bib-0006] Baenas, I. , Caravaca‐Sanz, E. , Granero, R. , Sánchéz, I. , Riesco, N. , Testa, G. , Fernández‐Aranda, F. , Treasure, J. , Jiménez‐Murcia, S. , & Fernández‐Aranda, F. (2020). COVID‐19 and eating disorders during confinement: Analysis of factors associated with resilience and aggravation of symptoms. European Eating Disorders Review, 28, 855–863. 10.1002/erv.2771 32815293 PMC7461472

[erv2861-bib-0007] Bhutani, S. , & Cooper, J. A. (2020). COVID‐19‐Related home confinement in adults: Weight gain risks and opportunities. Obesity, 28(9), 1576–1577. 10.1002/oby.22904 32428295 PMC7276847

[erv2861-bib-0008] Branley‐Bell, D. , & Talbot, C. (2020). Exploring the impact of the COVID‐19 pandemic and UK lockdown on individuals with experience of eating disorders. Journal of Eating Disorders, 8(44). 10.1186/s40337-020-00319-y PMC7444862 32874585

[erv2861-bib-0009] Brooks, S. K. , Webster, R. K. , Smith, L. E. , Woodland, L. , Wessely, S. , Greenberg, N. , & Rubin, G. J. (2020). The psychological impact of quarantine and how to reduce it: Rapid review of the evidence. The Lancet, 395, 912–920. 10.1016/S0140-6736(20)30460-8 PMC7158942 32112714

[erv2861-bib-0010] Castellini, G. , Cassioli, E. , Rossi, E. , Innocenti, M. , Gironi, V. , Sanfilippo, G. , Felciai, F. , Ricca, V. , & Ricca, V. (2020). The impact of COVID‐19 epidemic on eating disorders: A longitudinal observation of pre versus post psychopathological features in a sample of patients with eating disorders and a group of healthy controls. International Journal of Eating Disorders, 53, 1855–1862. 10.1002/eat.23368 PMC7461528 32856333

[erv2861-bib-0011] Cénat, J. M. , Blais‐Rochette, C. , Kokou‐Kpolou, C. K. , Noorishad, P. G. , Mukunzi, J. N. , McIntee, S. E. , Dalexis, R. D. , Goulet, M. A. , & Labelle, P. R. (2021). Prevalence of symptoms of depression, anxiety, insomnia, posttraumatic stress disorder, and psychological distress among populations affected by the COVID‐19 pandemic: A systematic review and meta‐analysis. Psychiatry Research, 295, 113599. 10.1016/j.psychres.2020.113599 33285346 PMC7689353

[erv2861-bib-0012] Clemmensen, C. , Petersen, M. B. , & Sørensen, T. (2020). Will the COVID‐19 pandemic worsen the obesity epidemic? Nature Reviews Endocrinology, 16(9), 469–470. 10.1038/s41574-020-0387-z PMC7342551 32641837

[erv2861-bib-0013] Cooper, M. , Reilly, E. E. , Siegel, J. A. , Coniglio, K. , Sadeh‐Sharvit, S. , Pisetsky, E. M. , & Anderson, L. M. (2020). Eating disorders during the Covid‐19 pandemic and quarantine: An overview of risks and recommendations for treatment and early intervention. The Journal of Treatment & Prevention (pp. 1–23). Advance online publication. 10.1080/10640266.2020.1790271 PMC7929530 32644868

[erv2861-bib-0014] Daly, M. , & Robinson, R. (2021). Longitudinal changes in psychological distress in the UK from 2019 to September 2020 during the COVID‐19 pandemic: Evidence from a large nationally representative study. Psychiatry Research, 300, 113920. 10.1016/j.psychres.2021.113920 33882397 PMC9755113

[erv2861-bib-0015] Fairburn, C. G. , & Beglin, S. J. (1994). Assessment of eating disorder psychopathology: Interview or self‐ report questionnaire? International Journal of Eating Disorders, 16, 363–370. 10.1016/j.brat.2003.07.008 7866415

[erv2861-bib-0016] Fernández‐Aranda, F. , Casas, M. , Claes, L. , Bryan, D. C. , Favaro, A. , Granero, R. , Gudiol, C. , Treasure, J. , Karwautz, A. , Le Grange, D. , Menchón, J. M. , Tchanturia, K. , & Treasure, J. (2020). COVID‐19 and implications for eating disorders. European Eating Disorders Review, 28, 239–245. 10.1002/erv.2738 32346977 PMC7267370

[erv2861-bib-0017] Fernández‐Aranda, F. , Munguía, L. , Mestre‐Bach, G. , Steward, T. , Etxandi, M. , Baenas, I. , Granero, R. , Sánchez, I. , Ortega, E. , Andreu, A. , Moize, V. L. , Fernández‐Real, J. M. , Tinahones, F. J. , Diegüez, C. , Frühbeck, G. , Le Grange, D. , Tchanturia, K. , Karwautz, A. , Zeiler, M. , … Jiménez‐Murcia, S. (2020). COVID Isolation Eating Scale (CIES): Analysis of the impact of confinement in eating disorders and obesity‐A collaborative international study. European Eating Disorders Review, 28(6), 871–883. 10.1002/erv.2784 32954595 PMC7537123

[erv2861-bib-0018] Furuya‐Kanamori, L. , Barendregt, J. J. , & Doi, S. A. R. (2018). A new improved graphical and quantitative method for detecting bias in meta‐analysis. International Journal of Evidence‐Based Healthcare, 16, 195–203. 10.1097/XEB.0000000000000141 29621038

[erv2861-bib-0019] Garner, D. (1991). Eating disorder inventory‐2. Psychological Assessment Resources.

[erv2861-bib-0020] Giel, K. E. , Schurr, M. , Zipfel, S. , Junne, F. , & Schag, K. (2021). Eating behaviour and symptom trajectories in patients with a history of binge eating disorder during COVID‐19 pandemic. European Eating Disorders Review, 29(4), 657–662. 10.1002/erv.2837 33955610 PMC8206923

[erv2861-bib-0021] Graell, M. , Morón‐Nozaleda, M. G. , Camarneiro, R. , Villaseñor, A. , Yáñez, S. , Muñoz, R. , Faya, M. , Miguélez‐Fernández, C. , Muñoz, M. , & Faya, M. (2020). Children and adolescents with eating disorders during COVID‐19 confinement: Difficulties and future challenges. European Eating Disorders Review, 28, 864–870. 10.1002/erv.2763 32729139

[erv2861-bib-0022] Holmes, E. A. , O'Connor, R. C. , Perry, V. H. , Tracey, I. , Wessely, S. , Arseneault, L. , Bullmore, E. , Christensen, H. , Cohen Silver, R. , Everall, I. , Ford, T. , John, A. , Kabir, T. , King, K. , Madan, I. , Michie, S. , Przybylski, A. K. , Shafran, R. , Sweeney, A. , … Bullmore, E. (2020). Multidisciplinary research priorities for the COVID‐19 pandemic: A call for action for mental health science. The Lancet Psychiatry, 7, 547–560. 10.1016/S2215-0366(20)30168-1 32304649 PMC7159850

[erv2861-bib-0023] Kaufman, E. A. , Xia, M. , Fosco, G. , Yaptangco, M. , Skidmore, C. R. , & Crowell, S. E. (2016). The difficulties in emotion regulation scale short form (DERS‐SF): Validation and replication in adolescent and adult samples. Journal of Psychopathology and Behavioral Assessment, 38(3), 443–455. 10.1007/s10862-015-9529-3 PMC12788809 41522882

[erv2861-bib-0024] Krishnamoorthy, Y. , Nagarajan, R. , Saya, G. K. , & Menon, V. (2020). Prevalence of psychological morbidities among general population, healthcare workers and COVID‐19 patients amidst the COVID‐19 pandemic: A systematic review and meta‐analysis. Psychiatry Research, 293, 113382. 10.1016/j.psychres.2020.113382 32829073 PMC7417292

[erv2861-bib-0025] Kroenke, K. , Spitzer, R. L. , & Williams, J. B. W. (2001). The PHQ‐9: Validity of a brief depression severity measure. Journal of General Internal Medicine, 16(9), 606–613. 10.1046/j.1525-1497.2001.016009606.x 11556941 PMC1495268

[erv2861-bib-0026] Kwok, S. , Adam, S. , Ho, J. H. , Iqbal, Z. , Turkington, P. , Razvi, S. , Le Roux, C. W. , Soran, H. , & Syed, A. A. (2020). Obesity: A critical risk factor in the COVID‐19 pandemic. Clinical Obesity, 10(6), e12403. 10.1111/cob.12403 32857454 PMC7460880

[erv2861-bib-0027] Lewis, Y. D. , Elran‐Barak, R. , Grundman‐Shem Tov, R. , & Zubery, E. (2021). The abrupt transition from face‐to‐face to online treatment for eating disorders: A pilot examination of patients’ perspectives during the COVID‐19 lockdown. Journal of Eating Disorders, 9, 31. 10.1186/s40337-021-00383-y 33673876 PMC7934980

[erv2861-bib-0028] Linardon, J. , Shatte, A. , Tepper, H. , & Fuller‐Tyszkiewicz, M. (2020). A survey study of attitudes toward, and preferences for, e‐therapy interventions for eating disorder psychopathology. International Journal of Eating Disorders, 53(6), 907–916. 10.1002/eat.23268 32239725

[erv2861-bib-0029] Lovibond, P. F. , & Lovibond, S. H. (1995). The structure of negative emotional states: Comparison of the depression anxiety stress scales (DASS) with the Beck depression and anxiety inventories. Behaviour Research and Therapy, 33(3), 335–343. 10.1016/0005-7967(94)00075-U 7726811

[erv2861-bib-0030] Luo, M. , Guo, L. , Yu, M. , Jiang, W. , & Wang, H. (2020). The psychological and mental impact of coronavirus disease 2019 (COVID‐19) on medical staff and general public ‐ a systematic review and meta‐analysis. Psychiatry Research, 291, 113190. 10.1016/j.psychres.2020.113190 32563745 PMC7276119

[erv2861-bib-0031] Machado, P. P. P. , Pinto‐Bastos, A. , Ramos, R. , Rodrigues, T. F. , Louro, E. , Goncalves, S. , Brandao, I. , & Vaz, A. (2020). Impact of COVID‐19 lockdown measures on a cohort of eating disorders patients. Journal of Eating Disorders, 8(1), 57. 10.1186/s40337-020-00340-1 33292539 PMC7831249

[erv2861-bib-0032] Marchitelli, S. , Mazza, C. , Lenzi, A. , Ricci, E. , Gnessi, L. , & Roma, P. (2020). Weight gain in a sample of patients affected by overweight/obesity with and without a psychiatric diagnosis during the Covid‐19 lockdown. Nutrients, 12(11), 3525. 10.3390/nu12113525 PMC7697678 33207742

[erv2861-bib-0033] Matheson, B. E. , Bohon, C. , & Lock, J. (2020). Family‐based treatment via videoconference: Clinical recommendations for treatment providers during COVID‐19 and beyond. International Journal of Eating Disorders, 53(7), 1142–1154. 10.1002/eat.23326 PMC7323318 32533799

[erv2861-bib-0034] McNamara, N. , Wakefield, J. , Cruwys, T. , Potter, A. , Jones, B. , & McDevitt, S. (2020). Family identification is a protective resource for people with eating disorders because it ameliorates feelings of loneliness. 10.31234/osf.io/xpjfh

[erv2861-bib-0035] Moher, D. , Shamseer, L. , Clarke, M. , Ghersi, D. , Liberati, A. , Petticrew, M. , Shekelle, P. , Stewart, L. A. , & Stewart, L. A. (2015). Preferred reporting items for systematic review and meta‐analysis protocols (PRISMA‐P) 2015 statement. Systematic Reviews, 4(1), 1. 10.1186/2046-4053-4-1 25554246 PMC4320440

[erv2861-bib-0036] Monteleone, A. M. , Cascino, G. , Marciello, F. , Abbate‐Daga, G. , Baiano, M. , Balestrieri, M. , Barone, E. , Monteleone, P. , Carpiniello, B. , Castellini, G. , Corrivetti, G. , De Giorgi, S. , Favaro, A. , Gramaglia, C. , Marzola, E. , Meneguzzo, P. , Monaco, F. , Oriani, M. G. , Pinna, F. , … Monteleone, P. (2021). Risk and resilience factors for specific and general psychopathology worsening in people with eating disorders during COVID‐19 pandemic: A retrospective Italian multicentre study. Eating and Weight Disorders, 10, 1–10. 10.1007/s40519-020-01097-x PMC7797193 33426630

[erv2861-bib-0037] Monteleone, A. M. , Marciello, F. , Cascino, G. , Abbate‐Daga, G. , Anselmetti, S. , Baiano, M. , Balestrieri, M. , Monteleone, P. , Bertelli, S. , Carpiniello, B. , Castellini, G. , Corrivetti, G. , De Giorgi, S. , Favaro, A. , Gramaglia, C. , Marzola, E. , Meneguzzo, P. , Monaco, F. , … Monteleone, P. (2021). The impact of COVID‐19 lockdown and of the following “re‐opening” period on specific and general psychopathology in people with eating disorders: The emergent role of internalizing symptoms. Journal of Affective Disorders, 285, 77–83. 10.1016/j.jad.2021.02.037 33636674 PMC9755808

[erv2861-bib-0038] Moreno, C. , Wykes, T. , Galderisi, S. , Nordentoft, M. , Crossley, N. , Jones, N. , Cannon, M. , Correll, C. U. , Byrne, L. , Carr, S. , Chen, E. , Gorwood, P. , Johnson, S. , Kärkkäinen, H. , Krystal, J. H. , Lee, J. , Lieberman, J. , López‐Jaramillo, C. , Männikkö, M. , Phillips, M. R. , Arango, C. , Vieta, E. , Vita, A. , & Arango, C. (2020). How mental health care should change as a consequence of the COVID‐19 pandemic. Lancet Psychiatry, 7(9), 813–824. 10.1016/S2215-0366(20)30307-2 32682460 PMC7365642

[erv2861-bib-0039] Murphy, R. , Calugi, S. , Cooper, Z. , & Dalle Grave, R. (2020). Challenges and opportunities for enhanced cognitive behaviour therapy (CBT‐E) in light of COVID‐19. Cognitive Behaviour Therapist, 13, e14. 10.1017/S1754470X20000161 34191937 PMC7264449

[erv2861-bib-0040] Niedzwiedz, C. L. , Green, M. J. , Benzeval, M. , Campbell, D. , Craig, P. , Demou, E. , Leyland, A. , Pearce, A. , Thomson, R. , Whitley, E. , & Katikireddi, S. V. (2021). Mental health and health behaviours before and during the initial phase of the COVID‐19 lockdown: Longitudinal analyses of the UK household longitudinal study. Journal of Epidemiology & Community Health, 75(3), 224–2020. 10.1136/jech-2020-215060 32978210 PMC7892383

[erv2861-bib-0041] Pedrosa, A. L. , Bitencourt, L. , Fróes, A. , Cazumbá, M. , Campos, R. , de Brito, S. , & Simões E Silva, A. C. (2020). Emotional, behavioral, and psychological impact of the COVID‐19 pandemic. Frontiers in Psychology, 11, 566212. 10.3389/fpsyg.2020.566212 33117234 PMC7561666

[erv2861-bib-0042] Pellegrini, M. , Ponzo, V. , Rosato, R. , Scumaci, E. , Goitre, I. , Benso, A. , Bo, S. , Crespi, C. , De Michieli, F. , Ghigo, E. , Broglio, F. , & Bo, S. (2020). Changes in weight and nutritional Habits in adults with obesity during the “Lockdown” period caused by the COVID‐19 virus emergency. Nutrients, 12, 2016. 10.3390/nu12072016 PMC7400808 32645970

[erv2861-bib-0043] Phillipou, A. , Meyer, D. , Neill, E. , Tan, E. , Lin Toh, W. , Van Rheenen, T. , & Rossell, S. (2020). Eating and exercise behaviors in eating disorders and the general population during the COVID‐19 pandemic in Australia: Initial results from the COLLATE project. International Journal of Eating Disorders, 53, 1158–1165. 10.1002/eat.23317 PMC7300745 32476163

[erv2861-bib-0044] Pierce, M. , Hope, H. , Ford, T. , Hatch, S. , Hotopf, M. , John, A. , Kontopantelis, E. , Webb, R. , Wessely, S. , McManus, S. , & Abel, K. M. (2020). Mental health before and during the COVID‐19 pandemic: A longitudinal probability sample survey of the UK population. Lancet Psychiatry, 7(10), 883–892. 10.1016/S2215-0366(20)30308-4 32707037 PMC7373389

[erv2861-bib-0045] Prati, G. , & Mancini, A. D. (2021). The psychological impact of COVID‐19 pandemic lockdowns: A review and meta‐analysis of longitudinal studies and natural experiments. Psychological Medicine, 51, 201–211. 10.1017/S0033291721000015 33436130 PMC7844215

[erv2861-bib-0046] Press, S. (2019). Stata statistical software: Release 16: StataCorp LLC.

[erv2861-bib-0047] Quittkat, H. L. , Hartmann, A. S. , Düsing, R. , Buhlmann, U. , & Vocks, S. (2019). Body dissatisfaction, importance of appearance and body appreciation in men and women over the lifespan. Frontiers in Psychiatry, 10, 864. 10.3389/fpsyt.2019.00864 31920737 PMC6928134

[erv2861-bib-0048] Richardson, C. , Pattona, M. , Phillips, S. , & Paslakis, G. (2020). The impact of the COVID‐19 pandemic on help‐seeking behaviors in individuals suffering from eating disorders and their caregivers. General Hospital Psychiatry, 67, 136–140. 10.1016/j.genhosppsych.2020.10.006 33129138 PMC10277602

[erv2861-bib-0049] Richter, D. , Riedel‐Heller, S. , & Zürcher, S. J. (2021). Mental health problems in the general population during and after the first lockdown phase due to the SARS‐cov‐2 pandemic: Rapid review of multi‐wave studies. Epidemiology and Psychiatric Sciences, 30, e27. 10.1017/S2045796021000160 33685551 PMC7985862

[erv2861-bib-0050] Robertson, M. , Duffy, F. , Newman, E. , Prieto Bravo, C. , Huseyin Ates, H. , & Sharpe, H. (2021). Exploring changes in body image, eating and exercise during the COVID‐19 lockdown: A UK survey. Appetite, 159, 105062. 10.1016/j.appet.2020.105062 33278549 PMC7711175

[erv2861-bib-0051] Rodgers, R. F. , Lombardo, C. , Cerolini, S. , Franko, D. L. , Omori, M. , Fuller‐Tyszkiewicz, M. , Linardon, J. , Courtet, P. , & Guillaume, S. (2020). The impact of the COVID‐19 pandemic on eating disorder risk and symptoms. International Journal of Eating Disorders, 53(7), 1166–1170. 10.1002/eat.23318 PMC7300468 32476175

[erv2861-bib-0052] Schlegl, S. , Maier, J. , Meule, A. , & Voderholzer, U. (2020). Eating disorders in times of the COVID‐19 pandemic. Results from an online survey of patients with anorexia nervosa. International Journal of Eating Disorders, 53, 1791–1800. 10.1002/eat.23374 PMC7461418 32841413

[erv2861-bib-0053] Schlegl, S. , Meule, A. , Favreau, M. , & Voderholzer, U. (2020). Bulimia nervosa in times of the COVID‐19 pandemic‐Results from an online survey of former inpatients. European Eating Disorders Review, 28(6), 847–854. 10.1002/erv.2773 32767858 PMC7436773

[erv2861-bib-0054] Shah, M. , Sachdeva, M. , & Johnston, H. (2020). Eating disorders in the age of COVID‐19. Psychiatry Research, 290, 113122. 10.1016/j.psychres.2020.113122 32480115 PMC7259905

[erv2861-bib-0055] Shevlin, M. , Butter, S. , McBride, O. , Murphy, J. , Gibson‐Miller, J. , Hartman, T. K. , Levita, L. , Mason, L. , Martinez, A. P. , McKay, R. , Stocks, T. , Bennett, K. , Hyland, P. , & Bentall, R. P. (2021). Refuting the myth of a 'tsunami' of mental ill‐health in populations affected by COVID‐19: Evidence that response to the pandemic is heterogeneous, not homogeneous. Psychological Medicine, 1–9. 10.1017/S0033291721001665 PMC8111207 33875044

[erv2861-bib-0056] Sidor, A. , & Rzymski, P. (2020). Dietary choices and Habits during COVID‐19 lockdown: Experience from Poland. Nutrients, 12(6), 1657. 10.3390/nu12061657 PMC7352682 32503173

[erv2861-bib-0057] Sisto, A. , Vicinanza, F. , Tuccinardi, D. , Watanabe, M. , Gallo, I. F. , D'Alessio, R. , Manfrini, S. , & Quintiliani, L. (2020). The psychological impact of COVID‐19 pandemic on patients included in a bariatric surgery program. Eating and Weight Disorders. 10.1007/s40519-020-00988-3 PMC7453189 32857287

[erv2861-bib-0058] Spitzer, R. L. , Kroenke, K. , Williams, J. B. , & Lowe, B. (2006). A brief measure for assessing generalized anxiety disorder the GAD‐7. Archives of Internal Medicine, 166, 1092–1097. 10.1001/archinte.166.10.1092 16717171

[erv2861-bib-0059] Taquet, M. , Luciano, S. , Geddes, J. R. , & Harrison, P. J. (2021). Bidirectional associations between COVID‐19 and psychiatric disorder: Retrospective cohort studies of 62 354 COVID‐19 cases in the USA. Lancet Psychiatry, 8(2), 130–140. 10.1016/S2215-0366(20)30462-4 33181098 PMC7820108

[erv2861-bib-0060] Termorshuizen, J. D. , Watson, H. J. , Thornton, L. M. , Borg, S. , Flatt, R. E. , MacDermod, C. M. , Harper, L. E. , Bulik, C. M. , Peat, C. M. , & Bulik, C. M. (2020). Early impact of COVID‐19 on individuals with self‐reported eating disorders: A survey of ∼1,000 individuals in the United States and The Netherlands. International Journal of Eating Disorders, 53(11), 1780–1790. 10.1002/eat.23353 32720399

[erv2861-bib-0061] Todisco, P. , & Donini, L. M. (2021). Eating disorders and obesity (ED&O) in the COVID‐19 storm. Eating and Weight Disorders, 26(3), 747–750. 10.1007/s40519-020-00938-z 32488728 PMC7265870

[erv2861-bib-0062] Vuillier, L. , May, L. , Greville‐Harris, M. , Surman, R. , & Moseley, R. L. (2021). The impact of the COVID‐19 pandemic on individuals with eating disorders: The role of emotion regulation and exploration of online treatment experiences. Journal of Eating Disorders, 9. 10.1186/s40337-020-00362-9 PMC7802411 33436064

[erv2861-bib-0064] Waller, G. , Pugh, M. , Mulkens, S. , Moore, E. , Mountford, V. A. , Carter, J. , Wicksteed, A. , Maharaj, A. , Wade, T. D. , Wisniewski, L. , Farrell, N. R. , Raykos, B. , Jorgensen, S. , Evans, J. , Thomas, J. J. , Osenk, I. , Paddock, C. , Bohrer, B. , Anderson, K. , … Smit, V. (2020). Cognitive‐behavioral therapy in the time of coronavirus: Clinician tips for working with eating disorders via telehealth when face‐to‐face meetings are not possible. International Journal of Eating Disorders, 53(7), 1132–1141. 10.1002/eat.23289 PMC7267365 32383530

[erv2861-bib-0065] Weissman, R. S. , Bauer, S. , & Thomas, J. J. (2020). Access to evidence‐based care for eating disorders during the COVID‐19 crisis. International Journal of Eating Disorders, 53(5), 369–646. 10.1002/eat.23279 PMC7267278 32338400

[erv2861-bib-0066] WHO . (2020). World Health Organization. Considerations for quarantine of individuals in the context of containment for coronavirus disease (COVID‐19)Retrieved from https://apps.who.int/iris/bitstream/handle/10665/331497/WHO‐2019‐nCoVIHR_Quarantine‐2020.2‐eng.pdf

[erv2861-bib-0067] Wu, T. , Jia, X. , Shi, H. , Niu, J. , Yin, X. , Xie, J. , & Wang, X. (2021). Prevalence of mental health problems during the COVID‐19 pandemic: A systematic review and meta‐analysis. Journal of Affective Disorders, 281, 91–98. 10.1016/j.jad.2020.11.117 33310451 PMC7710473

[erv2861-bib-0068] Xiong, J. , Lipsitz, O. , Nasri, F. , Lui, L. , Gill, H. , Phan, L. , Chen‐Li, D. , Iacobucci, M. , Ho, R. , Majeed, A. , & McIntyre, R. S. (2020). Impact of COVID‐19 pandemic on mental health in the general population: A systematic review. Journal of Affective Disorders, 277, 55–64. 10.1016/j.jad.2020.08.001 32799105 PMC7413844

[erv2861-bib-0069] Arroll, B. , Goodyear‐Smith, F. , Crengle, S. , Gunn, J. , Kerse, N. , Fishman, T. , Falloon, K. , & Hatcher, S. (2010). Validation of PHQ‐2 and PHQ‐9 to screen for major depression in the primary care population. Annals of Family Medicine, 8, 348–53. 10.1370/afm.1139 20644190 PMC2906530

[erv2861-bib-0070] Bernstein, D. P. , Stein, J. A. , Newcomb, M. D. , Walker, E. , Pogge, D. , Ahluvalia, T. , Stokes, J. , Handelsman, L. , Medrano, M. , Desmond, D. , & Zule, W. (2003). Development and validation of a brief screening version of the childhood trauma questionnaire. Child Abuse & Neglect, 27, 169–190. 10.1016/S0145-2134(02)00541-0 12615092

[erv2861-bib-0071] Blomquist, K. K. , Roberto, C. A. , Barnes, R. D. , White, M. A. , Masheb, R. M. , & Grilo, C. M. (2014). Development and validation of the eating loss of controlscale. Psychological Assessment, 26, 77–89. 10.1037/a0034729 24219700 PMC4021596

[erv2861-bib-0072] Bohn, K. , & Fairburn, C. G. (2008). The Clinical Impairment Assessment Questionnaire (CIA 3.0). In C. G. Fairburn (Ed.), Cognitive behavioral therapy for eating disorders. New York, NY: Guilford Press. p. 315–8.

[erv2861-bib-0073] Calugi, S. , Sartirana, M. , Milanese, C. , El Ghoch, M. , Riolfi, F. , & Dalle Grave, R. (2018). The clinical impairment assessment questionnaire: Validation in Italian patients with eating disorders. Eating and Weight Disorders, 23(5), 685–694. 10.1007/s40519-018-0477-2 29368290

[erv2861-bib-0074] Cloninger, C. R. (1999). The temperament and character inventory‐revised. St Louis, MO: Center for Psychobiology of Personality, Washington University.

[erv2861-bib-0075] Cohen, S. , Kamarck, T. , & Mermelstein, R. (1983). A global measure of perceived stress. Journal of Health and Social Behavior, 24, 386–96. 10.2307/2136404 6668417

[erv2861-bib-0076] Cowdrey, F. A. , & Park, R. J. (2011). Assessing rumination in eating disorders: principal component analysis of a minimally modified ruminative response scale. Eating Behaviors, 12, 321–4. 10.1016/j.eatbeh.2011.08.001 22051368

[erv2861-bib-0077] Derogatis, L . (1990). SCL‐90‐R. Administration, scoring and procedures manual—II for the revised version. Baltimore, MD: Clinical Psychometric Research.

[erv2861-bib-0078] Derogatis, L. R. , & Melisaratos, N. (1983). The brief symptom inventory: An introductory report. Psychological Medicine, 13(03), 595–605. 10.1017/S0033291700048017 6622612

[erv2861-bib-0079] Fairburn, C. G. , & Beglin, S. J. (1994). Assessment of eating disorders: Interview or self‐report questionnaire. International Journal of Eating Disorders, 16(4), 363‐370. 7866415

[erv2861-bib-0081] Foa, E. B. , Kozak, M. J. , Salkovskis, P. M. , Coles, M. E. , & Amir, N. (1998). The validation of a newobsessive‐compulsive disorder scale: The obsessive‐compulsive inventory. Psychological Assessment, 10, 206–214. 10.1037/1040-3590.10.3.206

[erv2861-bib-0082] Fraley, R. C. , Waller, N. G. , & Brennan, K. A. (2000). An item response theory analysis of self‐report measures of adult attachment. Journal of Personality and Social Psychology, 78, 350–365. 10707340 10.1037//0022-3514.78.2.350

[erv2861-bib-0083] Garner, D. M . (1991). Eating disorder inventory‐2: Professional manual. Psychological Assessment Resources.

[erv2861-bib-0084] Gearhardt, A. N. , Corbin, W. R. , & Brownell, K. D. (2016). Development of the Yale food addiction scale version 2.0. Psychology of Addictive Behaviors, 30(1), 113–121. 10.1037/adb0000136 26866783

[erv2861-bib-0085] Gormally, J. , Black, S. , Daston, S. , & Rardin, D. (1982). The assessment of binge eating severity among obese persons. Addictive Behaviors, 7, 47–55. 7080884 10.1016/0306-4603(82)90024-7

[erv2861-bib-0086] Hardt, J. A new questionnaire for measuring quality of life ‐ the Stark QoL. Health and Quality of Life Outcomes, 13, 174. 10.1186/s12955-015-0367-5 PMC4621869 26503323

[erv2861-bib-0087] Kaufman, J. , & Stoddard, J. (2020). Coronavirus impact scale. https://disasterinfo.nlm.nih.gov/search/?source=2587. Accessed 28 April 2020.

[erv2861-bib-0088] Kaufman, E. A. , Xia, M. , Fosco, G. , Yaptangco, M. , Skidmore, C.R. , & Crowell, S. E. (2016). Thedifficulties in emotion regulation scale short form (DERS‐SF): validation andreplication in adolescent and adult samples. Journal of Psychopathology and Behavioral Assessment, 38, 443–55. 10.1007/s10862-015-9529-3 PMC12788809 41522882

[erv2861-bib-0089] Kroenke, K. , Spitzer, R. L. , & Williams, J. B. W. (2001). The PHQ‐9: Validity of a brief depressionseverity measure. Journal of General Internal Medicine, 16, 606–613. 10.1046/j.1525-1497.2001.016009606.x 11556941 PMC1495268

[erv2861-bib-0090] Kroenke, K. , Spitzer, R. L. , Williams, J. B. , & Lowe, B. (2009). An ultra‐brief screeningscale for anxiety and depression: The PHQ–4. Psychosomatics, 50(6), 613–621. 19996233 10.1176/appi.psy.50.6.613

[erv2861-bib-0091] Lovibond, S. H. , & Lovibond, P. F. (1995). Manual for the Depression Anxiety Stress Scales. (2nd ed.). Psychology Foundation.

[erv2861-bib-0092] McLaughlin, E. (2014). The EAT‐16: Validation of a shortened form of the Eating Attitudes Test. https://digitalrepository.unm.edu/cgi/viewcontent.cgi‐article=1093&context=psy_etds

[erv2861-bib-0093] Plummer, F. , Manea, L. , Trepel, D. , & McMillan, D. (2016). Screening for anxietydisorders with the GAD‐7 and GAD‐2: a systematic review and diagnostic metaanalysis. General Hospital Psychiatry, 39, 24–31. 26719105 10.1016/j.genhosppsych.2015.11.005

[erv2861-bib-0094] Postmes, T. , Haslam, S. A. , & Jans, L. (2013). A single‐item measure of social identification:Reliability, validity, and utility. British Journal of Social Psychology, 52(4), 597‐617. 10.1111/bjso.12006 23121468

[erv2861-bib-0095] Rush, A. J. , Trivedi, M. H. , Ibrahim, H. M. , Carmody, T. J. , Arnow, B. , Klein, D. N. , Markowitz, J. C. , Ninan, P. T. , Kornstein, S. , Manber, R. , Thase, M. E. , Kocsis, J. H. , & Keller, M. B. (2003). The 16‐Item Quick Inventory of Depressive Symptomatology (QIDS), clinician rating (QIDS‐C), and self‐report (QIDS‐SR): a psychometric evaluation in patients with chronic major depression. Biological Psychiatry, 54(5), 573‐83. 10.1016/S0006-3223(02)01866-8 12946886

[erv2861-bib-0096] Shapiro, D. H. Jr . (1994). Shapiro control inventory (SCI) manual. http://controlresearch.net/shapiro‐control‐inventory‐manual.html Accessed 4 May 2020.

[erv2861-bib-0097] Spitzer, R. L. , Kroenke, K. , Williams, J. B. W. , & Löwe, B. (2006). A brief measure for assessing generalized anxiety disorder: The GAD‐7. Archives of Internal Medicine, 166, 1092–1097. 10.1001/archinte.166.10.1092 16717171

[erv2861-bib-0098] Tennant, R. , Hiller, L. , Fishwick, R. , Platt, S. , Joseph, S. , Weich, S. , Parkinson, J. , Secker, J. , & Stewart‐Brown, S. (2007). The Warwick‐Edinburgh Mental Well‐being Scale (WEMWBS): development and UK validation. Health and Quality of Life Outcomes, 5, 63. 10.1186/1477-7525-5-63 18042300 PMC2222612

[erv2861-bib-0099] United States Department of Agriculture (USDA) . (2012). U.S. Household FoodSecurity Survey Module: Six‐Item Short Form. U.S. Department of Agriculture, Economic Research Service. https://www.ers.usda.gov/media/8279/ad2012.pdf

[erv2861-bib-0100] Vaglio, J. , Conard, M. , Poston, W. S. , O’Keefe, J. , Haddock, C. K. , House, J. , & Spertus, J. A. (2004). Testing the performance of the ENRICHD social support instrument incardiac patients. Health and Quality of Life Outcomes. 10.1186/1477-7525-2-2 PMC434528 15142277

[erv2861-bib-0101] Varni, J. W. , Seid, M. , & Kurtin, P. S. (2001). PedsQL 4.0: reliability and validity of the pediatric quality of life inventory version 4.0 generic core scales inhealthy and patient populations. Medical Care, 39(8), 800‐812. 11468499 10.1097/00005650-200108000-00006

[erv2861-bib-0102] Weiss, D. S. , & Marmar, C. R. (1997). The impact of event scale‐revised. In J. P. Wilson & T. M. Keane (Eds.), Assessing psychological trauma and PTSD (pp. 399–411). Guilford Press.

[erv2861-bib-0103] Weathers, F. W. , Litz, B. T. , Keane, T. M. , Palmieri, P. A. , Marx, B. P. , & Schnurr, P. P. (2013). The PTSD Checklist for DSM‐5 (PCL‐5) – Standard [Measurement instrument]. https://www.ptsd.va.gov/

[erv2861-bib-0104] Whiteside, S. P. , Lynam, D. R. , Miller, J. D. , & Reynolds, S. K. (2005). Validation of the UPPS impulsive behavior scale: a four factor model of impulsivity. European Journal of Personality, 74, 574‐559. 10.1002/per.556

