# Supplementary material for: Effects of COVID‐19 lockdown on eating disorders and obesity: A systematic review and meta‐analysis
Source: Eur Eat Disord Rev. 2021 Aug 30;29(6):826–41. doi: 10.1002/erv.2861 (PMC8652707; doi:10.1002/erv.2861)
Supplement: Supplementary file 1 — Supporting Information S1 [file ERV-29-826-s001.docx]

Supplementary materials Eating disorders and COVID

**Supplementary Table 1**

*Quality assessment of included studies on Eating Disorders*

| **Authors, date** | **Study design** | **Preprint** | **Representativeness of the sample** | **Sample size** | **Non-respondents** | **Ascertainment of the exposure** | **Confounders** | **Assessment of the outcome** | **Statistical analyses** | **Independent longitudinal assessment** | **Total score** | **Risk of bias** |
| --- | --- | --- | --- | --- | --- | --- | --- | --- | --- | --- | --- | --- |
| Baenas et al., 2020 | cross sectional | No | 1 | 1 | 0 | 1 | 2 | 0 | 1 | 0 | 6 | Medium |
| Branley-Bell & Talbot, 2020 | mixed, cross sectional | No | 0 | 1 | 0 | 1 | 0 | 0 | 1 | 0 | 3 | High |
| Castellini et al., 2020 | longitudinal, case control | No | 1 | 1 | 1 | 1 | 1 | 1 | 1 | 1 | 8 | Low |
| Fernández-Aranda et al., 2020b | cross sectional | No | 1 | 1 | 0 | 1 | 1 | 1 | 1 | 0 | 6 | Medium |
| Graell et al., 2020 | cross sectional | No | 1 | 1 | 0 | 1 | 0 | 1 | 0 | 0 | 4 | High |
| Machado et al., 2020 | longitudinal | no | 1 | 0 | 0 | 1 | 0 | 1 | 1 | 1 | 5 | Medium |
| McNamara et al., 2020 | cross sectional | yes | 0 | 1 | 0 | 1 | 1 | 1 | 1 | 0 | 5 | Medium |
| Monteleone et al., 2021a | cross sectional | No | 1 | 1 | 0 | 1 | 2 | 1 | 1 | 0 | 7 | Low |
| Monteleone et al., 2021b | cross sectional | No | 1 | 1 | 0 | 1 | 2 | 1 | 1 | 0 | 7 | Low |
| Phillipou et al., 2020 | case control | No | 0 | 1 | 0 | 1 | 0 | 1 | 1 | 0 | 4 | High |
| Quittkat et al., 2020 | cross sectional | No | 0 | 1 | 0 | 1 | 0 | 1 | 1 | 0 | 4 | High |
| Richardson et al., 2020 | mixed, cross sectional | No | 1 | 1 | 0 | 1 | 0 | 0 | 1 | 0 | 4 | High |
| Robertson et al., 2021 | cross sectional | No | 0 | 0 | 0 | 1 | 0 | 0 | 1 | 0 | 2 | High |
| Schlegl et al., 2020a | cross sectional | No | 1 | 1 | 0 | 1 | 0 | 0 | 0 | 0 | 3 | High |
| Schlegl et al., 2020b | cross sectional | No | 1 | 1 | 0 | 1 | 0 | 0 | 0 | 0 | 3 | High |
| Termorshuizen et al., 2020 | mixed, cross sectional | No | 0 | 1 | 0 | 1 | 1 | 0 | 1 | 0 | 4 | High |
| Vuillier et al., 2021 | mixed, cross sectional | No | 0 | 1 | 0 | 1 | 2 | 1 | 1 | 0 | 6 | Medium |

**Supplementary Table2**

*Quality assessment of included studies on Obesity*

| **Authors, date** | **Study design** | **Preprint** | **Representativeness of the sample** | **Sample size** | **Non-respondents** | **Ascertainment of the exposure** | **Confounders** | **Assessment of the outcome** | **Statistical analyses** | **Independent longitudinal assessment** | **Total score** | **Risk of bias** |
| --- | --- | --- | --- | --- | --- | --- | --- | --- | --- | --- | --- | --- |
| Abawi et al, 2020 | mixed, longitudinal | No | 1 | 1 | 0 | 1 | 1 | 1 | 0 | 1 | 6 | Medium |
| Ahmed, 2020 | cross sectional | No | 1 | 1 | 0 | 1 | 1 | 1 | 0 | 1 | 6 | Medium |
| Almandoz et al., 2020 | cross sectional | No | 1 | 1 | 0 | 1 | 1 | 1 | 1 | 0 | 6 | Medium |
| Athanasiadis et al. 2020 | cross sectional | No | 1 | 1 | 0 | 0 | 2 | 1 | 1 | 0 | 6 | Medium |
| Fernández-Aranda et al., 2020b | cross sectional | No | 1 | 0 | 0 | 1 | 1 | 1 | 1 | 0 | 5 | Medium |
| Marchitelli et al.,2020 | cross sectional | No | 1 | 1 | 1 | 1 | 2 | 1 | 1 | 0 | 8 | Low |
| Pellegrini et al., 2020 | longitudinal | No | 1 | 1 | 1 | 1 | 2 | 0 | 1 | 1 | 7 | Low |
| Sidor et al., 2020 | case control | No | 0 | 1 | 0 | 1 | 0 | 0 | 0 | 0 | 2 | High |
| Sisto et al., 2020 | cross sectional | No | 1 | 1 | 0 | 1 | 2 | 1 | 1 | 0 | 7 | Low |
| Waledziak et al., 2020 | cross sectional | No | 0 | 1 | 0 | 1 | 0 | 0 | 0 | 0 | 2 | High |

**Supplementary Table 3**

*Summary of characteristics of included studies on Eating Disorders*

| **Authors, date** | **Country, time** | **Data collection** | **N** | **Population** | **Female, n (%)*** | **Age, n/ mean*** | **Diagnosis , n (%)*** | **BMI** | **Exposure definition** | **Exposure / mean**** | **Outcome definition** | **Outcome assessment** | **Other predictors** | **Predictors assessment** |
| --- | --- | --- | --- | --- | --- | --- | --- | --- | --- | --- | --- | --- | --- | --- |
| Baenas et al., 2020 | Spain, April, 2020 | Telephone survey | 74 | Adult | 71 (95.9) | 32.12 | AN: 19 (25.7); BN: 12 (16.2); BED: 10 (13.5); OSFED: 33 (44.6) | nr | COVID-19 lockdown | 2 | EDs worsening | ad hoc non-validated questionnaire | Age; BMI; ED symptoms; food addiction; psychological symptoms; self-directedness; COVID related distress | EDI-2; SCL-90-R; YFAS-2; TCI-R; ad hoc non-validated questionnaire |
| Branley-Bell &Talbot, 2020 | Spain, April, 2020 | Social media | 129 | Adult | 121 (93.8) | 29.27 | nr | nr | COVID-19 lockdown | 2 | EDs worsening; EDs recovery | ad hoc non-validated questionnaire | Mental wellbeing; stress; social support; control; rumination | SWEMWBS; PSS; ESSI;  SCI; RRS-ED |
| Castellini et al., 2020 | Italy, April 2020 | Face to face and video interview, and online survey | 74 | Adult | 74 (100) | 31.74 | AN: 37 (50); BN: 37 (50) | 22.31 | COVID-19 lockdown | 2 | EDs symptoms; stress; BMI | ad hoc non validated questionnaire; BSI; EDE-Q; IES-R | Age; education; childhood trauma; attachment insecurity | CTQ-SF; ECR-R |
| Fernández-Aranda et al., 2020b | Spain, June–July2020 | nr | 87 | Adult | AN: 49 (89.1); BN: 17 (94.4); OSFED: 12 (85.7) | AN: 24.16; BN: 31.50; OSFED: 36.86 | AN: 55 (63.2); BN: 18 (20.7); OSFED: 14 (16.1) | Before pandemic, AN: 18.25; BN: 24.13; OSFED: 23.40 | COVID-19 lockdown | 4-5 | EDs symptoms;BMI | CIES | Diagnosis | Ad hoc questionnaire |
| Graell et al., 2020 | Spain, March – May 2020 | Clinical records, face to face, telephone, and video interview | 365 | Children and adolescents | 321 (88) | Day hospital: 13.18; outpatient service: 14.74 | ARFID: 48 (13.2); AN: 255 (69.9); BN: 26 (7); OSFED: 37 (10) | Before pandemic, day hospital:18.66; outpatient service:19.55 | COVID-19 lockdown | 1-2 | EDs symptoms; psychological symptoms | Clinical records and ad hoc questionnaire | none | na |
| Machado et al., 2020 | Portugal, April – May 2020 | Clinical records, telephone interview, and online questionnaire | 43 | Adults | 41(95.3) | 27.60 | AN: 20 (46.5);  BN: 14 (32.6);  BED: 2 (4.7);  OSFED: 7 (16.3) | Before pandemic, 20.53 | COVID-19 lockdown; Coronavirus related distress | 2-3; CIS | EDs symptoms; emotion dysregulation; impulsivity; BMI | EDE-Q; CIA; DERS-SF;  UPPS-P | none | na |
| McNamara et al., 2020 | UK, USA, Ireland  Individuals with self-reported EDs: April – June 2020 | Online survey | 157 | Adults | nr | nr | AN: 82 (52);  BN: 17 (11);  OSFED: 20 (13); BED: 8 (5); ARFID: 3 (2) | nr | Loneliness | Three-Item Loneliness Scale  Service users: mean 6.86 (SD 1.89)  Individuals with self-reported EDs: 7.06 (1.79) | EDs symptoms; EDs COVID-19 related impact | EAT-16; ad hoc questionnaire | Age, gender, diagnosis, family identification  Anxiety | Postmes et al.’s (2013) single-item group  identification measure  DASS-21 |
| Monteleone et al., 2021a | Italy, June 2020 | Online survey | 312 | Adults | 300 (96.2) | 29.19 | AN: 179 (57.4); BN: 48 (15.4); BED: 22 (7.05); OSFED: 63 (20.2). | AN (17.25); BN (22.13); BED (33.54); OSFED (20.43) | COVID-19 lockdown | 3 | EDs symptoms; depression; anxiety; OC symptoms; PTSD symptoms | EDI-2; PHQ-9; GAD-7. OCI; PCL-5 | Age; illness duration; diagnosis | Clinical records |
| Monteleone et al., 2021b | Italy, June 2020 | Online survey | 312 | Adults | 300 (96.2) | 29.19 | AN: 179 (57.4); BN: 48 (15.4); BED: 22 (7.05); OSFED: 63 (20.2). | AN (17.25); BN (22.13); BED (33.54); OSFED (20.43) | Loneliness | Before pandemic: AN: mean 4.17 (SD3.1)  Other EDs: 3.41 (2.67) | EDs symptoms; depression; anxiety; OC symptoms; PTSD symptoms | EDI-2; PHQ-9; GAD-7. OCI; PCL-5 | Age; illness duration; diagnosis; economic loss; psychotherapy; satisfaction with therapy; satisfaction with family, friends, and sentimental relationships | Ad hoc questionnaire |
| Phillipou et al., 2020 | Australia, April 2020 | Online survey | 180 | Adults | 172(95.6) | 30.47 | AN: 88 (48.9); BN: 23(12.8); BED: 6(3.3); OSFED: 4 (2.2); unspecified: 68 (37.8) | nr | COVID-19 lockdown | 2 | EDs symptoms; depression; anxiety | EDE-Q; DASS-21 | none | na |
| Quittkat et al., 2020 | Germany, April 2020 | Online survey | 62 | Adults | nr | nr | nr | nr | COVID-19 lockdown | 2 | EDs symptoms; stress | EDE-Q; PHQ |  |  |
| Richardson et al., 2020 | Canada, April 2020 | Telephone interview | 439 | Adults | 353 (80.4) | age 26+: 134 (30.5%); ages 15–19: 136 (28.7%) | AN: 84 (19); BN: 44 (10); BED 64 (15); OSFED: 8 (2); ARFID: 4(1); other: 235 (53) | nr | COVID-19 lockdown | 2 | EDs symptoms | ad hoc questionnaire | none | na |
| Robertson et al., 2021 | UK, May-June2020 | Online survey | 35 | Adults | 35 (100) | 35 | AN: 20 (57); BN: 5 (15);  BED: 1 (3);OSFED: 1 (3);  ARFID: 1 (3);  Multiple diagnoses: 7 (20) | nr | COVID-19 lockdown | 3-4 | EDs symptoms; stress | ad hoc questionnaire; PHQ-4 | none | na |
| Schlegl et al., 2020a | Germany, May 2020 | Online survey | 159 | Adolescents (47) and adults (112) | 159 (100) | 22.42 | AN | 17.83 (2.43); < 18.5: 92 (60.1%); ≥ 18.5: 61 (38.4) | COVID-19 lockdown | 3 | EDs symptoms | ad hoc questionnaire | none | na |
| Schlegl et al., 2020b | Germany, May 2020 | Online survey | 55 | Adolescents (2) and adults (53) | 55 (100) | 24.42 | BN | 23.62 | COVID-19 lockdown | 3 | EDs symptoms | ad hoc questionnaire | none | na |
| Termorshuizen et al., 2020 | US, Netherlands, April - May 2020 | Online survey | US: 511; Netherlands:510 | Adolescents and adults | US: 495 (97); Netherlands:506 (99) | US: 16–21: 63 (12); 22–29: 230 (45); 30–39: 133 (26); 40–49: 57 (11); 50+: 27 (5)  Netherlands: 16–21: 127 (25); 22–29: 219 (43); 30–39: 111 (22); 40–49: 27 (5); 50+: 26 (5) | US: AN:318 (62); BN: 178 (35); BED: 156 (31); Atypical AN: 100 (20); OSFED: 122 (24); Purging disorder: 38 (7); ARFID: 34 (7); NES: 20 (4).  Netherlands:AN: 347 (68); BN: 117 (23); BED: 60 (12); Atyp. AN: 103 (20); OSFED: 70 (14); Purging disorder: 9 (2);ARFID: 2 (0.4); NES: 5 (1) | nr | COVID-19 lockdown | 2-3 | EDs symptoms | ad hoc questionnaire | Gender;age;day of study enrolment | na |
| Vuillier et al., 2021 | UK, nr | Online survey | 207 | Adults | 131 (63) | nr | AN: 91 (44); BN: 46 (22); BED: 44 (21); OSFED: 26 (13) | nr | COVID-19 lockdown  Change in social support, physical activity, food availability, and treatment;  exposure to triggering messages; negative emotions; disruption to routine and living situation;  physical health concerns | Ad hoc questionnaire  Change in social support: mean 55.9 (SD 31.7), physical activity69.8 (30.1), food av. 55.0 (34.5), treatment69.8 (30.1);  exposure to triggering messages 57.0 (34.9); negative emotions 71.8 (26.5); disruption to routine71.2 (27.9) and living situation62.3 (27.6);  physical health concerns52.4 (34.8) | EDs symptoms; depression; anxiety; emotional dysregulation | EDE-Q; DASS; GAD-7;  DERS-SF | none | na |

*Note*. * when frequency not available they were calculated from percentages; ** for COVID-19 lockdown, n months from February 2020; AN = Anorexia Nervosa; ARFID = Avoidant/ Restrictive Food Intake Disorder; BED = Binge Eating Disorder; BMI = Body Mass Index; BN = Bulimia Nervosa; BSI = Brief Symptom Inventory; CIA = Clinical Impairment Assessment; CIES = COVID Isolation Eating Scale; CIS = Coronavirus Impact Scale; CTQ-SF = Childhood Trauma Questionnaire–Short Form; DASS = Depression and Anxiety Stress Scale; DERS-SF = Difficulties in Emotion Regulation Scale; EAT-16 = Eating Attitudes Test 16 items; ECR-R = Experiences in Close Relationships–Revised; EDE-Q = Eating Disorder Examination Questionnaire; EDI-2 = Eating Disorders Inventory-2; ESSI = ENRIHD Social Support Instrument; GAD-7 = Generalized Anxiety Disorder 7; IES-R = Impact of Event Scale-Revised; na = not applicable; NES = Night-Eating Syndrome; nr = not reported; OCI = Obsessive-Compulsive Inventory; OSFED = Otherwise Specified Feeding and Eating Disorder; PHQ-9 = Patient Health Questionnaire 9; PCL-5 = PTSD Checklist forDSM-5; RRS-ED = Rumination Response Scale for Eating Disorders; SCI = Shapiro Control Inventory; SCL-90-R = Symptom checklist-90-revised; SES = Socio Economic Status; SF-36 = Short Form 36 items; SWEMWBS = Short Warwick-Edinburgh mental wellbeing scale; TCI-R = Temperament and Character Inventory- Revised; UPPS-P = Impulsive Behavior Scale; YFAS-2 = Yale Food Addiction Scale version 2.0

**Supplementary Table 4**

*Summary of characteristics of included studies on Obesity*

| **Authors, date** | **Country, time** | **Data collection** | **N** | **Population** | **Female, n (%)*** | **Age, n/ mean*** | **Diagnosis , n (%)*** | **BMI** | **Exposure definition** | **Exposure, n / mean**** | **Outcome definition** | **Outcome assessment** | **Other predictors** | **Predictors assessment** |
| --- | --- | --- | --- | --- | --- | --- | --- | --- | --- | --- | --- | --- | --- | --- |
| Abawi et al., 2020 | Netherlands, April 2020 | Telephone interview, clinical records | 75 | Children | 39(52) | 10.5 | Severe obesity: 75 (100) | nr | COVID-19 lockdown | 2 | COVID-19 related anxiety; quality of life | Ad hoc questionnaire; PedsQL | Gender; age; BMI | Ad hoc questionnaire |
| Ahmed, 2020 | Iraq, April - June 2020 | Face-to- face interview | 765 | Adults | 302 (39.5) | < 20: 9 (1);  21–30: 349 (46);31–40: 208 (27);  41–50: 120 (16);51–60: 39 (5);  61–70: 30 (4) | nr [service users of bariatric clinic] | nr | COVID-19 lockdown | 2-4 | Wight gain; emotional state | Ad hoc questionnaire; modified SF-36 questionnaire | Gender | Ad hoc questionnaire |
| Almandoz et al., 2020 | US, April - May 2020 | Online survey, clinical records | 123 | Adults | 107 (87) | 51.2 | nr [service users of bariatric clinic] | 40.2 | COVID-19 lockdown | 2-3 | Weight loss; exercising; depression; anxiety | Ad hoc questionnaire; QIDS-SR | Ethnicity  . | Ad hoc questionnaire |
| Athanasiadis et al. 2020 | US, nr | Online survey | 208 | Adults | 179(86) | 48.9 | nr [service users of bariatric clinic] | nr | COVID-19 lockdown; social distancing | nr | Eating symptoms; depression; anxiety | Modified version of the ELOCS, PHQ-9, and GAD-2 | Gender; age; ethnicity; SES; weight change; length of lockdown | Ad hoc questionnaire |
| Fernández-Aranda et al., 2020b | Spain, June–July2020 | nr | 34 | Adult | 26(76.5) | 48.82 | nr [service users of bariatric clinic] | Before pandemic: 41.15 | COVID-19 lockdown | 4-5 | EDs symptoms; psychological symptoms; BMI | CIES | none | na |
| Marchitelli et al., 2020 | Italy, April – May2020 | Online survey | 110 | Adult | without a psychiatric diagnosis: 42/ 63 (66.7);  with a psychiatric diagnosis:36/ 47 (76.6) | without a psychiatric diagnosis: 47.24;  with a psychiatric diagnosis: 46.38 | without a psychiatric diagnosis: overweight3 (4.8); obesity  class I8 (12.7); class II26 (41.3);class III26 (41.3);  with a psychiatric diagnosis: overweight 3 (6.4); obesity  class I 6 (12.8); class II 12 (25.5); class III 26 (55.3) | without a psychiatric diagnosis: 40.19;  with a psychiatric diagnosis: 39.88 | COVID-19 lockdown; psychiatric disorders | 2-3; GHQ-12 | EDs symptoms; weight gain; depression; anxiety; emotion dysregulation; risk perception; social support | BES;DASS-21; DERS; ad hoc questionnaire | Gender; age; education; BMI; use of medications; use of psychotherapy | Ad hoc questionnaire |
| Pellegrini et al., 2020 | Italy, April 2020 | Online survey | 150 | Adults | 116 (77.3) | 47.9 | nr[service users of obesity clinic] | Before pandemic: 34.4 | COVID-19 lockdown | 2 | Weight gain; BMI | Clinical records | Gender; age; education; BMI; anxiety/depression; boredom/solitude; eating behaviours | Ad hoc questionnaire |
| Sidor et al., 2020 | Poland, April 2020 | Online survey | 94 | Adults | 83 (88.3) | 33.5 | nr | nr | COVID-19 lockdown | 2 | Weight gain | Ad hoc questionnaire | none | na |
| Sisto et al., 2020 | Italy, April 2020 | Online survey | 434 | Adults | 379 (87.3) | 45.6 | nr [service users of bariatric clinic, pre-bariatric surgery: 59 (13.6); post-bariatric: 375 (86.4)] | nr | COVID-19 related change in eating behaviour, emotions, and resilience | Ad hoc questionnaire | EDs symptoms; depression; anxiety | Ad hoc questionnaire; DASS-21 | Gender; age | Ad hoc questionnaire |
| Waledziak et al., 2020 | Poland, April 2020 | Online survey | 800 | Adults | 703 (88) | Median: 39 | nr [service users of bariatric clinic, pre-bariatric surgery: 258(32); post-bariatric:542 (68)] | Median: 34.26 | COVID-19 lockdown | 2 | EDs symptoms; depression; anxiety | Ad hoc questionnaire | none | na |

*Note*. * when frequency not available they were calculated from percentages; ** for COVID-19 lockdown, n months from February 2020; BES = Binge Eating Scale; BMI = Body Mass Index; CIES = COVID Isolation Eating Scale; DASS = Depression and Anxiety Stress Scale; DERS-SF = Difficulties in Emotion Regulation Scale; ELOCS = Patient Health Questionnaire; GAD-7 = Generalized Anxiety Disorder 7; na = not applicable; nr = not reported; PedsQL = Paediatric Quality of Life inventory;PHQ-9 = Patient Health Questionnaire 9; QIDS-SR= 16-item Quick Inventory of Depressive Symptomatology; SES = Socio Economic Status; SF-36 = Short Form 36 items

**Supplementary Figures 1-2**

*Forrest and Funnel Plot of change in BMI from the pre-pandemic to the pandemic time in Eating Disorders*

**
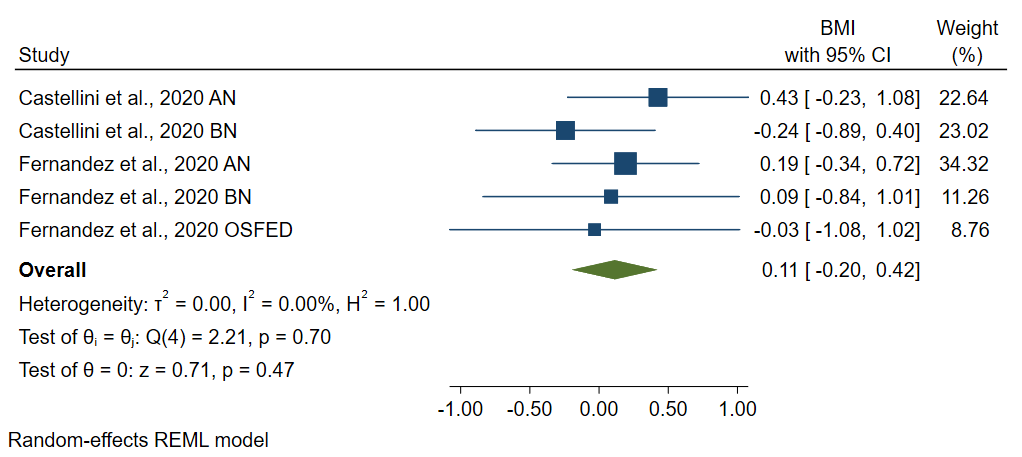

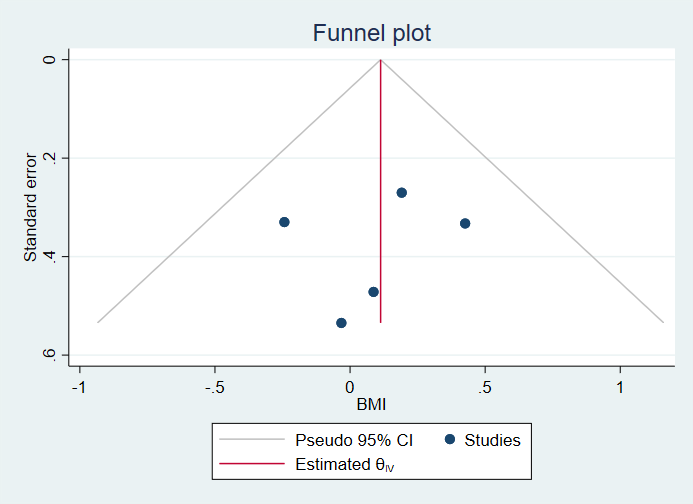
**

**Supplementary Figures 3-4**

*Forrest plot of change in ED symptom severity and impact of ED symptoms from the pre-pandemic to the pandemic time in Eating Disorders*

**
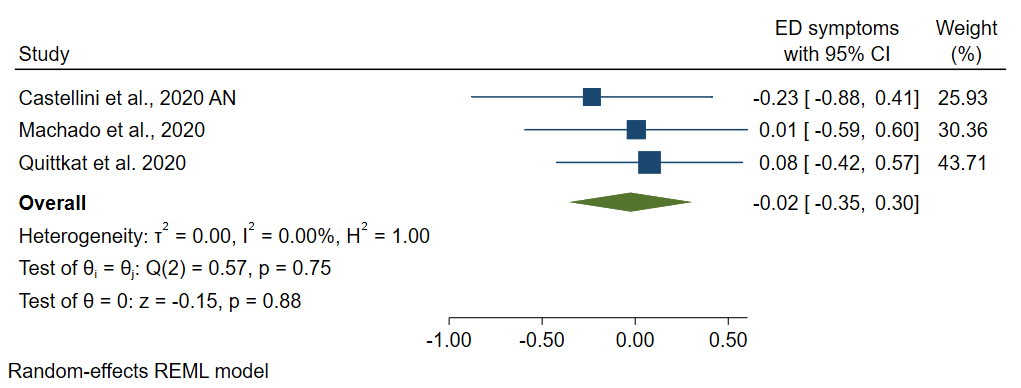

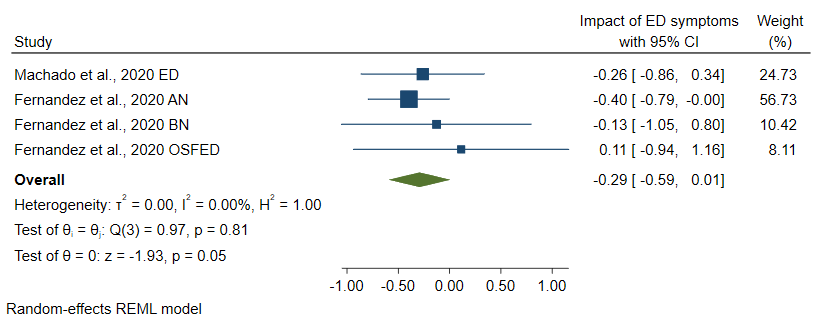
**

**Supplementary Figure 5**

*Forrest plot of change in binging from the pre-pandemic to the pandemic time in Eating Disorders*

**
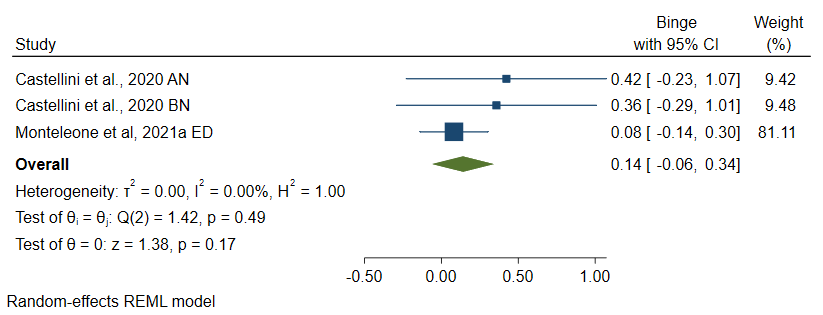
**

**Supplementary Figures 6-7**

*Forrest and Funnel Plot of change in Mental Health Symptoms from the pre-pandemic to the pandemic time in Eating Disorders*

**
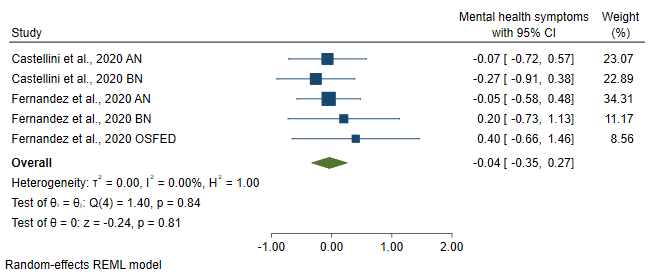

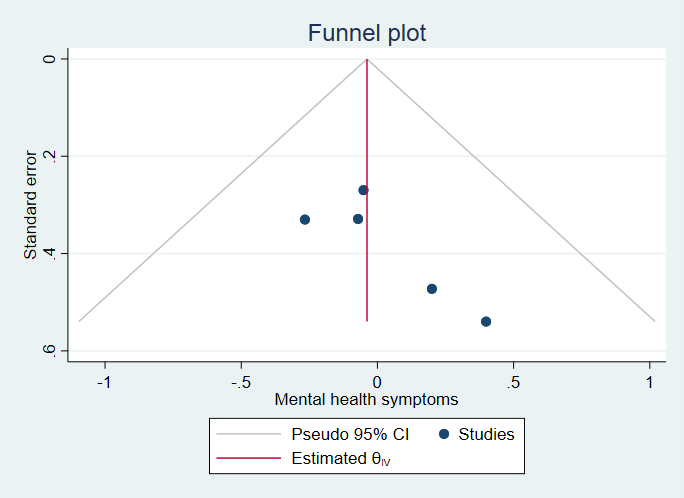
**

**References for measures for Eating Disorders symptoms and mental health symptoms**

Arroll, B., Goodyear-Smith, F., Crengle, S., Gunn, J., Kerse, N., Fishman, T., Falloon, K., & Hatcher, S. (2010). Validation of PHQ-2 and PHQ-9 to screen for major depression in the primary care population. *Annals of Family Medicine,* **8**, 348–53. <https://doi.org/10.1370/afm.1139>

Bernstein, D. P., Stein, J. A., Newcomb, M. D., Walker, E., Pogge, D., Ahluvalia, T., Stokes, J., Handelsman, L., Medrano, M., Desmond, D., & Zule, W. (2003). Development and validation of a brief screening version of the childhood trauma questionnaire. *Child Abuse & Neglect*, **27**, 169–190. <https://doi.org/10.1016/S0145-2134(02)00541-0>

Blomquist, K.K., Roberto, C.A., Barnes, R.D., White, M.A., Masheb, R.M., & Grilo, C.M. (2014). Development and validation of the eating loss of controlscale*. Psychological Assessment*, **26**, 77–89.<https://doi.org/10.1037/a0034729>

Bohn, K., & Fairburn, C.G.(2008). The Clinical Impairment Assessment Questionnaire (CIA 3.0). In: Fairburn CG, editor. *Cognitive behavioral therapy for eating disorders*. New York,NY: Guilford Press. p. 315–8.

Calugi, S., Sartirana, M., Milanese, C., El Ghoch, M., Riolfi, F., & Dalle Grave, R. (2018). The clinical impairment assessment questionnaire: Validation in Italian patients with eating disorders. *Eating and Weight Disorders*, **23**(5), 685–694. <https://doi.org/10.1007/s40519-018-0477-2>

Cloninger, C. R. (1999). The temperament and character inventory-revised. St Louis, MO: Center for Psychobiology of Personality, Washington University.

Cohen, S., Kamarck, T., & Mermelstein, R. (1983). A global measure of perceived stress. *Journal of Health and Social Behavior*,**24**, 386–96. <https://doi.org/10.2307/2136404>

Cowdrey, F.A., & Park, R.J. (2011). Assessing rumination in eating disorders: principal component analysis of a minimally modified ruminative response scale. *Eating Behaviors,* **12**, 321–4. <https://doi.org/10.1016/j.eatbeh.2011.08.001>

Derogatis, L. (1990). SCL-90-R. Administration, scoring and procedures manual—II for the revised version. Baltimore, MD: Clinical Psychometric Research.

Derogatis, L. R., & Melisaratos, N. (1983). The brief symptom inventory: An introductory report. *Psychological Medicine*, **13**(03), 595–605.<https://doi.org/10.1017/S0033291700048017>

Fairburn, C. G., & Beglin, S. J. (1994). Assessment of eating disorders: Interview or self-report questionnaire. *International Journal of Eating Disorders*, **16**(4), 363-370.

Fernández-Aranda, F., Munguía, L., Mestre-Bach, G., Steward, T., Etxandi, M., Baenas, I., Granero, R., Sánchez, I., Ortega, E., Andreu, A., Moize, V. L., Fernández-Real, J. M., Tinahones, F. J., Diegüez, C., Frühbeck, G., Le Grange, D., Tchanturia, K., Karwautz, A., Zeiler, M., Favaro, A., … Jiménez-Murcia, S. (2020). COVID Isolation Eating Scale (CIES): Analysis of the impact of confinement in eating disorders and obesity-A collaborative international study. *European eating disorders Review: the journal of the Eating Disorders Association*, **28**(6), 871–883. <https://doi.org/10.1002/erv.2784>

Foa, E.B., Kozak, M.J., Salkovskis, P.M., Coles, M.E., &Amir, N. (1998). The validation of a newobsessive-compulsive disorder scale: The obsessive-compulsive inventory. *Psychological Assessment*, **10**, 206–214. <https://doi.org/10.1037/1040-3590.10.3.206>

Fraley, R. C., Waller, N. G., & Brennan, K. A. (2000). An item response theory analysis of self-report measures of adult attachment. *Journal of Personality and Social Psychology*, **78**, 350–365.

Garner, D.M. (1991). Eating disorder inventory-2: Professional manual. *Psychological Assessment Resources*, Odessa.

Gearhardt, A. N., Corbin, W. R., & Brownell, K. D. (2016). Development of the Yale food addiction scale version 2.0. *Psychology of Addictive Behaviors*, **30**(1), 113–121. <https://doi.org/10.1037/adb0000136>

Gormally, J., Black, S., Daston, S., & Rardin, D. (1982). The assessment of binge eating severity among obese persons. *Addictive Behaviors*, **7**, 47–55.

Hardt, J. (2015). A new questionnaire for measuring quality of life - the Stark QoL. *Health and Quality of Life Outcomes,* **13**, 174. https://doi.org/10.1186/s12955-015-0367-5

Kaufman, J., & Stoddard, J. (2020). Coronavirus impact scale. https://disasterinfo.nlm.nih.gov/search/?source=2587. Accessed 28 April 2020

Kaufman, E.A., Xia, M., Fosco, G., Yaptangco, M., Skidmore, C.R., & Crowell, S.E. (2016). Thedifficulties in emotion regulation scale short form (DERS-SF): validation andreplication in adolescent and adult samples. *Journal of Psychopathology and Behavioral Assessment*, **38**, 443–55. <https://doi.org/10.1007/s10862-015-9529-3>

Kroenke, K., Spitzer, R.L., & Williams, J.B.W. (2001). The PHQ-9: Validity of a brief depressionseverity measure.*Journal of General Internal Medicine.***16**, 606–613. <https://doi.org/10.1046/j.1525-1497.2001.016009606.x>

Kroenke, K., Spitzer, R. L., Williams, J. B., & Lowe, B. (2009). An ultra-brief screeningscale for anxiety and depression: The PHQ–4. *Psychosomatics*, **50**(6), 613–621.

Lovibond, S.H., & Lovibond, P.F. (1995). Manual for the Depression Anxiety Stress Scales(2nd ed.). Sydney: Psychology Foundation.

McLaughlin, E. (2014). The EAT-16: Validation of a shortened form of the Eating AttitudesTest. Retrieved from<https://digitalrepository.unm.edu/cgi/viewcontent.cgi?article=1093&context=psy_etds>

Plummer, F., Manea, L., Trepel, D., & McMillan, D. (2016). Screening for anxietydisorders with the GAD-7 and GAD-2: a systematic review and diagnostic metaanalysis. *General Hospital Psychiatry*, **39**, 24–31.

Postmes, T., Haslam, S. A., & Jans, L. (2013). A single-item measure of social identification:Reliability, validity, and utility. *British Journal of Social Psychology*, **52**(4), 597-617.

Rush, A.J., Trivedi, M.H., Ibrahim, H.M., Carmody, T.J., Arnow, B., Klein, D.N., Markowitz, J.C., Ninan, P.T., Kornstein, S., Manber, R., Thase, M.E., Kocsis, J.H., & Keller, M.B. (2003). The 16-Item Quick Inventory of Depressive Symptomatology (QIDS), clinician rating (QIDS-C), and self-report (QIDS-SR): a psychometric evaluation in patients with chronic major depression. *Biological Psychiatry*, **54**(5), 573-83. <https://doi.org/10.1016/S0006-3223(02)01866-8>.

Shapiro Jr. DH. (1994). Shapiro control inventory (SCI) manual. http://controlresearch.net/shapiro-control-inventory-manual.html Accessed 4 May2020

Spitzer, R.L., Kroenke, K., Williams, J.B.W., & Löwe, B. (2006). A brief measure for assessing generalized anxiety disorder: The GAD-7. *Archives of Internal Medicine*, **166**, 1092–1097. <https://doi.org/10.1001/archinte.166.10.1092>

Tennant, R., Hiller, L., Fishwick, R., Platt, S., Joseph, S., Weich, S., Parkinson, J., Secker, J., & Stewart-Brown, S. (2007). The Warwick-Edinburgh Mental Well-being Scale (WEMWBS): development and UK validation. *Health and Quality of Life Outcomes,* **5**, 63. https://doi.org/10.1186/1477-7525-5-63

United States Department of Agriculture (USDA) (2012). *U.S. Household FoodSecurity Survey Module: Six-Item Short Form*. Washington, DC: U.S. Department of Agriculture, Economic Research Service. Available from<https://www.ers.usda.gov/media/8279/ad2012.pdf>

Vaglio, J., Conard, M., Poston, W.S., O’Keefe, J., Haddock, C.K., House, J., & Spertus, J.A. (2004). Testing the performance of the ENRICHD social support instrument incardiac patients. *Health and Quality of Life Outcomes*. https://doi.org/10.1186/1477-7525-2-2

Varni, J.W., Seid, M., & Kurtin, P.S. (2001). PedsQL 4.0: reliability and validity of the pediatric quality of life inventory version 4.0 generic core scales inhealthy and patient populations. *Medical Care*, **39**(8), 800-812.

Weiss, D. S., & Marmar, C. R. (1997). The impact of event scale-revised. In J. P. Wilson & T. M. Keane (Eds.), *Assessing psychological trauma and PTSD* (pp. 399–411). New York, NY: Guilford Press.

Weathers, F. W., Litz, B. T., Keane, T. M., Palmieri, P. A., Marx, B. P., & Schnurr, P. P. (2013). The PTSD Checklist for DSM-5 (PCL-5) – Standard [Measurement instrument]. Availablefrom https://www.ptsd.va.gov/

Whiteside, S.P., Lynam, D.R., Miller, J.D., & Reynolds, S.K. (2005). Validation of the UPPS impulsive behavior scale: a four factor model of impulsivity. *European Journal of Personality*, **74**, 574-559. https://doi.org/10.1002/per.556
